# Supplementary material for: Harnessing macrophage-drug conjugates for allogeneic cell-based therapy of solid tumors via the TRAIN mechanism
Source: Nat Commun. 2025 Feb 4;16:1327. doi: 10.1038/s41467-025-56637-9 (PMC11790938; doi:10.1038/s41467-025-56637-9)
Supplement: Supplementary file 1 — Supplementary Information [file 41467_2025_56637_MOESM1_ESM.pdf]

Description of supplementary information

Taciak B., Białasek M., Kubiak M., (…), Kucharzewska P., Rygiel T., Król M.:

Harnessing Macrophage-Drug Conjugates for Allogeneic Cell-Based Therapy of Solid Tumors via the TRAIN Mechanism

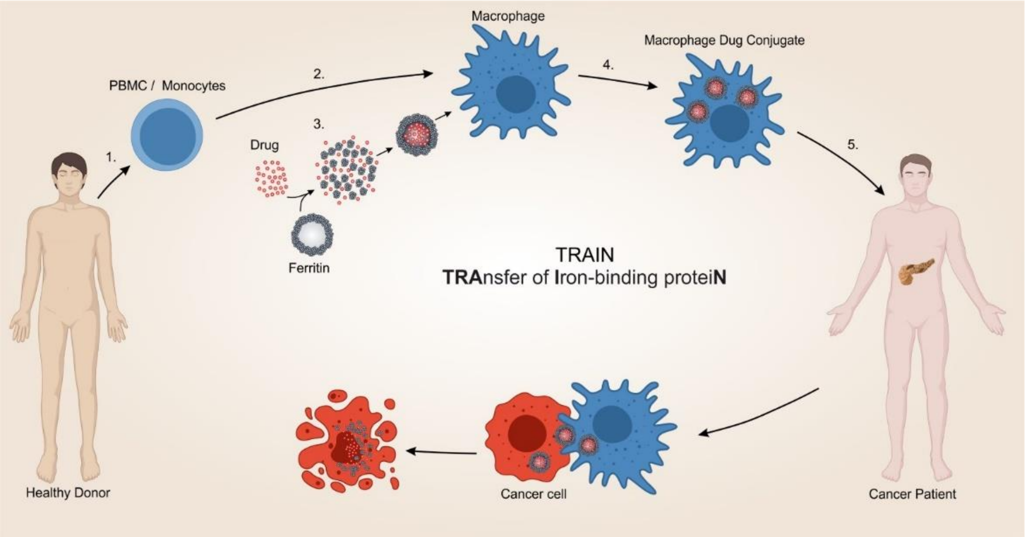

Supplementary Figure 1. Steps for MDC generation and therapeutic delivery for targeted tumor treatment. The MDC generation and human therapy will involve the following steps: (1) isolation of monocytes from the peripheral blood, (2) *ex vivo* differentiation of monocytes into macrophages, (3) complexation of the desired drug with ferritin cage, (4) loading of the drug-complexed ferritin into the macrophages to form the MDC, and (5) targeting the tumor upon administration the MDC to the patient where MDC transfer the drug into the surrounding cancer cells, leading to their death. Created in BioRender. Taciak, B. (2024) https://BioRender.com/m57d544.

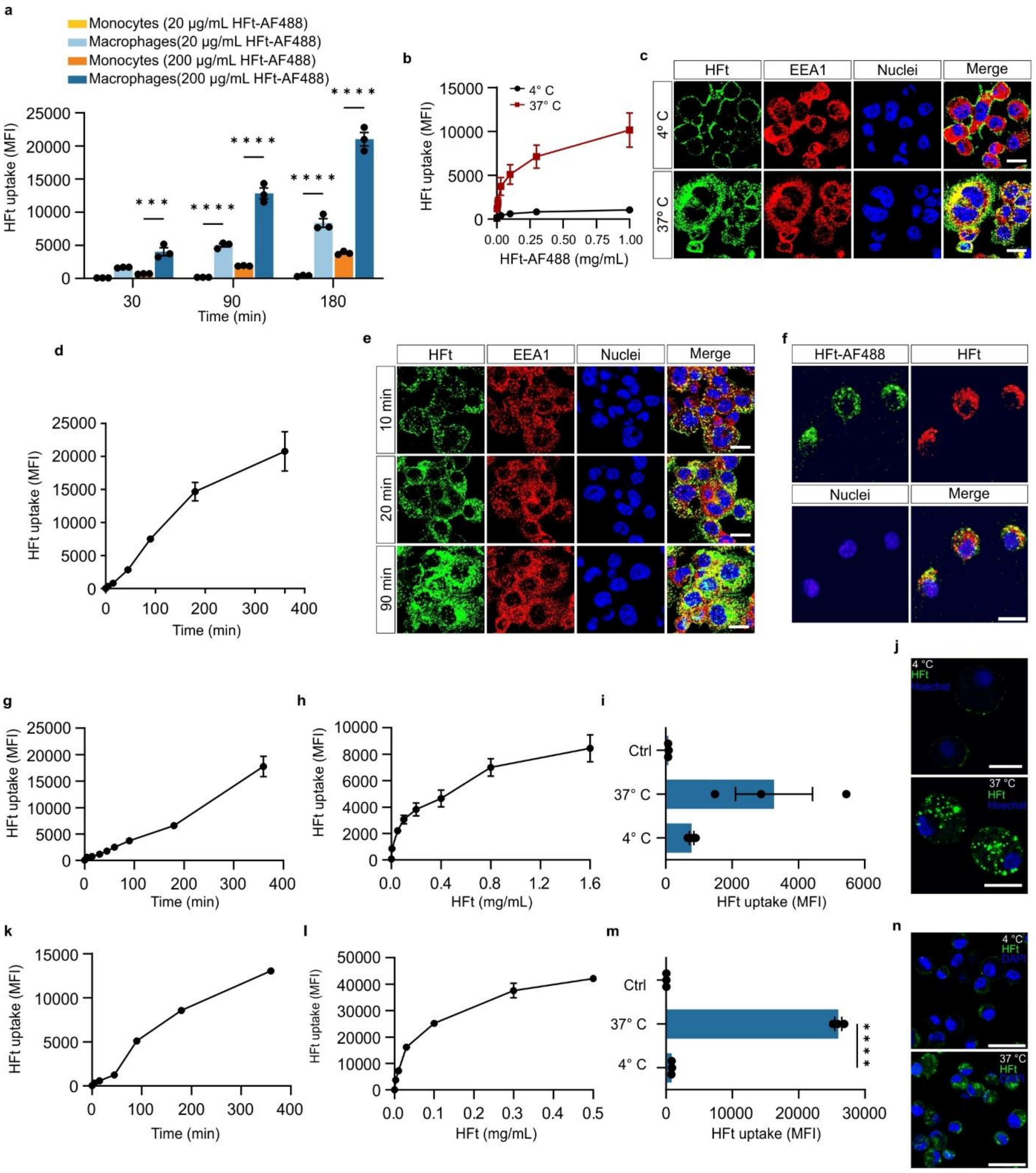

Supplementary Figure 2. Time and concentration-dependent uptake of H-ferritin in human and mouse macrophages. a Flow cytometry of HFt-AF488 uptake by THP-1 monocytes and macrophages at indicated concentrations and time points at 37 °C. **b** Flow cytometry of HFt-AF488 uptake by THP-1 macrophages at various concentrations (3–1000 μg/ml) for 30 min at 4 °C or 37 °C. **c** Representative confocal microscopy images of HFt-AF488 (100 μg/ml) uptake by THP-1 macrophages for 30 min at 4 °C or 37 °C. Cells were stained with anti-EEA1 antibody and nuclei with Hoechst 33342; merged images show colocalization (yellow) at 37 °C. Scale bar: 20 μm. **d** Flow cytometry of HFt-AF488 (100 μg/ml) uptake by THP-1 macrophages at indicated time points at 37 °C. **e** Representative confocal microscopy images of HFt-AF488 (50 μg/ml, green) uptake by THP-1 macrophages at different time points at 37 °C. Cells were stained with anti-EEA1 antibody and nuclei with Hoechst 33342. Scale bar: 20 μm. **f** Representative confocal microscopy images of HFt-AF488 uptake by THP-1 macrophages for 30 min at 37 °C. Cells were stained with anti-HFt and nuclei with Hoechst 33342; merged images show colocalization (yellow foci). Scale bar: 20 μm. **g, k** Flow cytometry of HFt-AF488 (100 μg/ml) uptake by hiPSC-derived macrophages (**g**) and BMDMs (**k**) at indicated time points at 37 °C. **h**, **l** Flow cytometry of HFt-AF488 uptake by hiPSC-derived macrophages (**h**) and BMDMs (**l**) at indicated concentrations for 30 min at 37 °C. **i**, **m** Flow cytometry of HFt-AF488 (100 μg/ml) uptake by hiPSC-derived macrophages (**i**) and BMDMs (**m**) for 30 min at 4 °C or 37 °C. **j**, **n** Representative confocal microscopy images of HFt-AF488 (100 μg/ml) uptake by hiPSC-derived macrophages (**j**) and BMDMs (**n**) for 30 min at 4 °C or 37 °C. Nuclei stained with Hoechst 33342 and DAPI. Scale bar: 20 μm. Flow cytometry data is a mean fluorescence intensity (MFI) of HFt-AF488. Values

represent mean ± SEM from n = 3 independent replicates. Two-way ANOVA with Tukey's post-hoc test used in **a**; one-way ANOVA with Tukey's post-hoc tests in **i**, **m**. For all panels, ***$P \leq 0.001$, ****$P \leq 0.0001$. Source data are provided as a Source Data file.

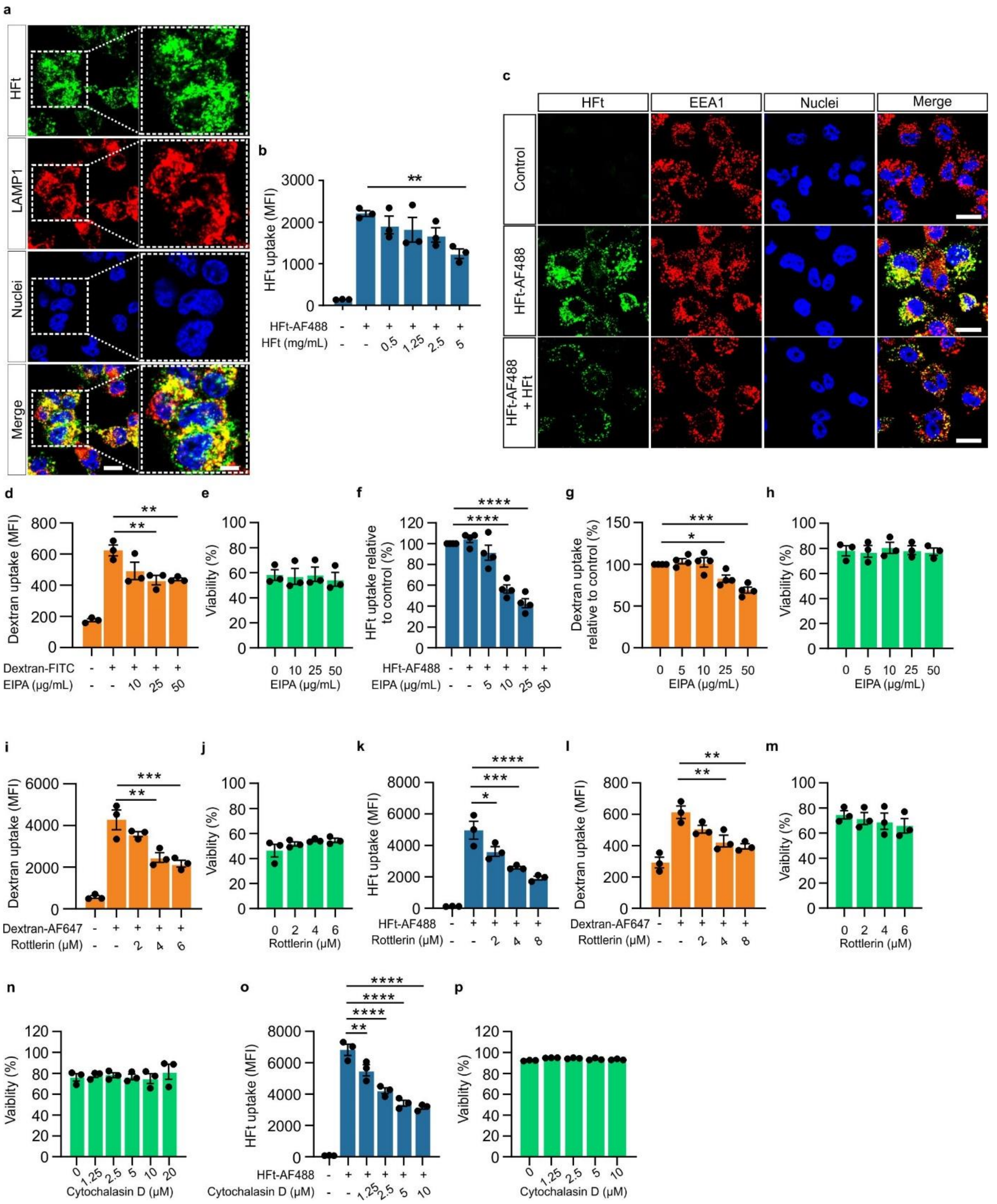

Supplementary Figure 3. H-ferritin enters human macrophages through the endocytic route. a Representative confocal microscopy images of HFt-AF488 uptake by hMDM (50 μg/ml, 60 min, 37 °C). Cells were fixed, stained with anti-LAMP1 antibody and nuclei with Hoechst 33342; merged images show colocalization (yellow foci). Scale bar: 20 μm (10 μm in zoomed region). **b–c** Flow cytometry (**b**) and representative confocal microscopy images (**c**) of HFt-AF488 uptake (10 μg/ml) by THP-1 macrophages in the absence or presence unlabeled HFt (5 mg/ml) for 30 min at 37 °C. In **c**, fixed cells were stained with anti-EEA1 antibody and nuclei with Hoechst 33342 (blue). Scale bar: 20 μm. **d**, **i** Flow cytometry of Dextran-FITC/Alexa647 (100 μg/ml) uptake by hMDM with or without EIPA (**d**) or rottlerin (**i**) for 30 min at 37 °C. **e**, **h**, **j**, **m**, **n**, **p** Viability of hMDM (**e**, **j**, **n**) and THP-1 macrophages (**h**, **m**, **p**) after 1 h treatment with EIPA (**e**, **h**), rottlerin (**j**, **m**), or cytochalasin D (**n**, **p**) assessed by flow cytometry; untreated controls used; data as % of live cells.**f–g**, **k–l**, **o** Flow cytometry of HFt-AF488 (50 μg/ml) (**f**, **k**, **o**) and Dextran-AF647 (100 μg/ml) (**g**, **l**) uptake by THP-1 macrophages within 30 min at 37 °C with or without EIPA (**f–g**), rottlerin (**k–l**), or cytochalasin D (**o**). Flow cytometry data is a mean fluorescence intensity (MFI) or MFI relative to control condition (f-g) of fluorescently labeled ligands; values represent mean ± SEM from n = 3 donors (hMDM) or n = 3 independent replicates (THP-1). One-way

ANOVA with Dunnett's post-hoc tests was used for statistical analysis. For all panels, *P ≤ 0.05, **P ≤ 0.01, ***P ≤ 0.001, ****P ≤ 0.0001. Source data are provided as a Source Data file.

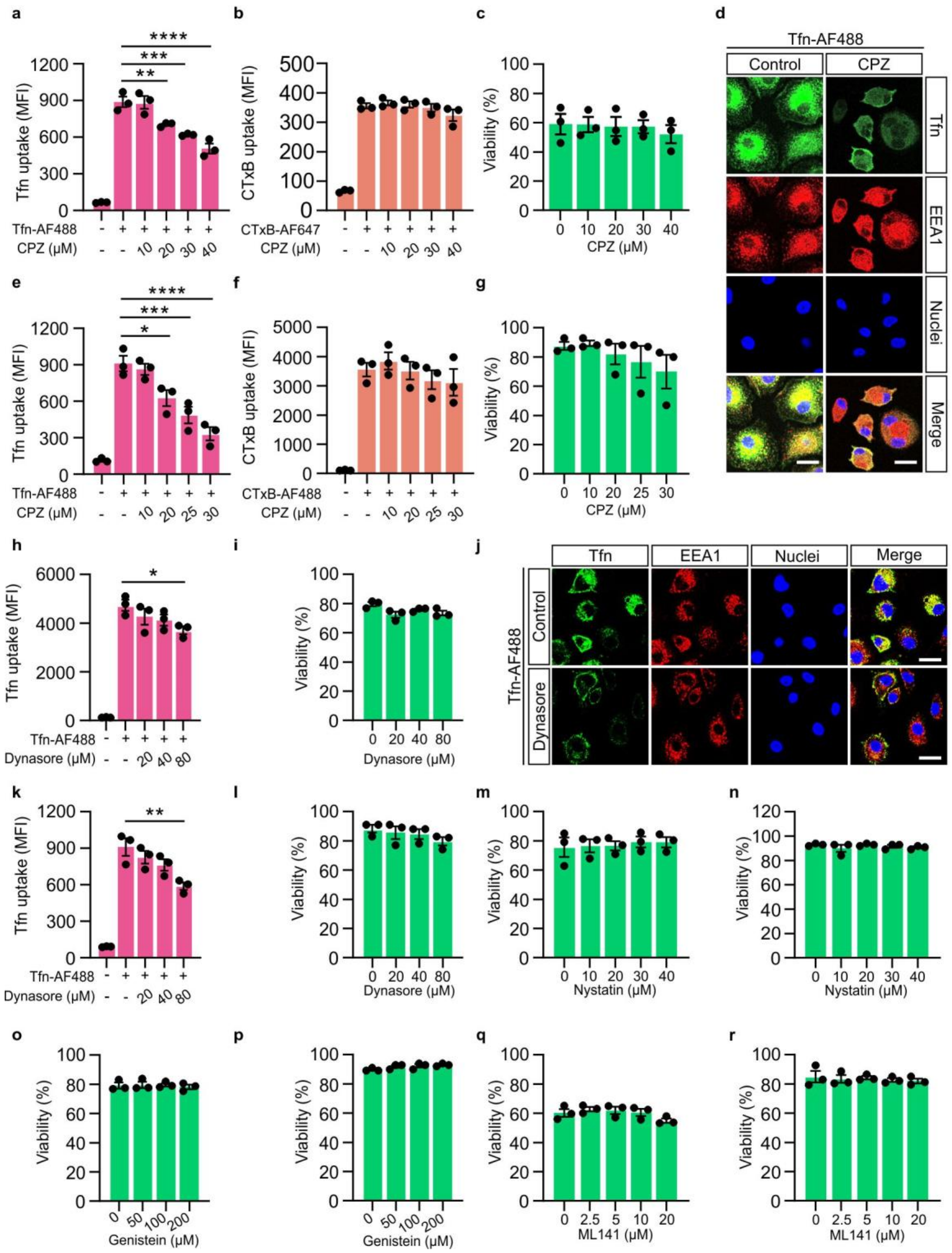

Supplementary Figure 4. H-ferritin enters human macrophages via clathrin-dependent endocytosis. a–b Flow cytometry of Tfn-AF488 (25 μg/ml) (**a**) and CTxB-AF647 (1 μg/ml) (**b**) uptake by hMDM in the absence or presence of chlorpromazine (CPZ) for 30 min at 37 °C; mean ± SEM from n = 3 independent donors or replicates. **c, g** Viability of hMDM (**c**) and THP-1 macrophages (**g**) after 1 h CPZ treatment; data are % live cells; mean ± SEM from n = 3 donors (hMDM) and 3 independent replicates (THP-1). **d** Representative confocal microscopy images of Tfn-AF488 (25 μg/ml) uptake by hMDM with or without CPZ (30 μM) for 30 min at 37 °C; cells stained with anti-EEA1 and nuclei with Hoechst 33342. Scale bar: 20 μm. **e–f** Flow cytometry of Tfn-AF488 (25 μg/ml) (**e**) and CTxB-AF647 (**f**) uptake by THP-1 macrophages with or without CPZ for 30 min at 37 °C; mean ± SEM from n = 3 independent donors or replicates. **h, k** Flow cytometry of Tfn-AF488 uptake by hMDM (**h**) and THP-1 macrophages (**k**) with or without dynasore at indicated concentrations for 15 min at 37 °C; mean ± SEM from n = 3 independent donors or replicates. **i, l** Viability of hMDM (**i**) and THP-1 macrophages (**l**) after 1 h dynasore treatment; data are % live cells; mean ± SEM from n = 3 independent donors or replicates. **j** Representative confocal microscopy images of Tfn-AF488 (25 μg/ml) uptake by hMDM with or without dynasore (80 μM) for 30 min at 37 °C; cells stained as in **d**. Scale bar: 20 μm. **m, o, q** hMDM viability after 1 h treatment with nystatin (**m**), genistein (**o**), or ML-141 (**q**); untreated cells as controls; data are % live cells; mean ± SEM

from n = 3 donors. **n, p, r** THP-1 macrophage viability after 1 h treatment with nystatin (**n**), genistein (**p**), or ML-141 (**r**); untreated cells as controls. data are % live cells; mean ± SEM from n = 3 independent replicates. The viability of cells was assessed by flow cytometry. One-way ANOVA with Dunnett's post-hoc test used. For all panels, *P ≤ 0.05, **P ≤ 0.01, ***P ≤ 0.001, ***P ≤ 0.0001. Source data are provided as a Source Data file.

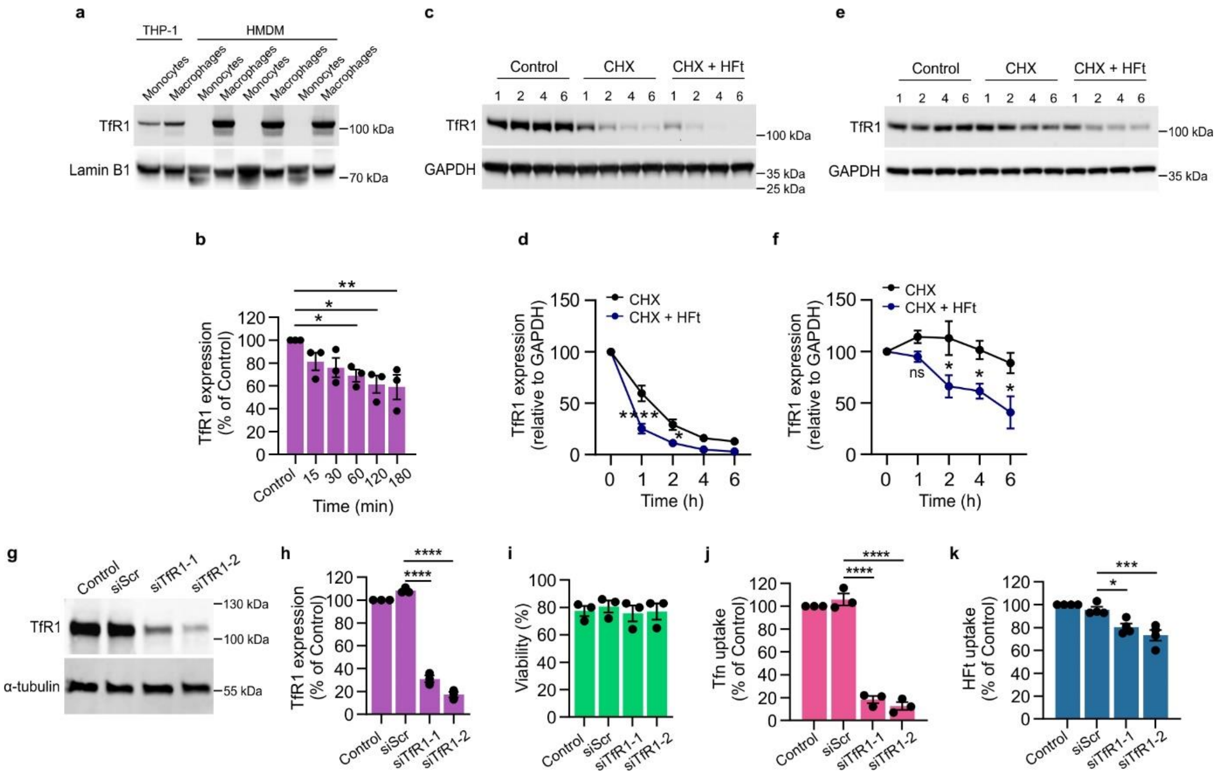

Supplementary Figure 5. H-ferritin uptake in human macrophages partially depends on TfR1. **a** Western blot of TfR1 expression in THP-1 monocytes/macrophages and human peripheral blood monocytes/hMDM. **b** Flow cytometry of TfR1 surface staining in THP-1 macrophages stimulated with HFt (200 µg/ml) for indicated time points at 37 °C; untreated cells (Control) used for comparison. Data (% of TfR1 expression in control cells) are mean ± SEM from n = 3 independent replicates. **c, e** Western blot of TfR1 expression in hMDM (c) or THP-1 macrophages (e) untreated (Control), pretreated with cycloheximide (CHX, 20 µg/ml) for 1 h before HFt stimulation (200 µg/ml) (CHX+HFt) for indicated times at 37 °C; cells treated only with CHX were controls. **d, f** Quantitative analysis of relative TfR1 expression in hMDM (d) and THP-1 macrophages (f) shown in c and e, respectively. Data are mean ± SEM from n = 3 (d-CHX) or n = 3 (d-CHX + HFt, f) independent replicates. **g** Western blot showing TfR1 in untransfected THP-1 macrophages (Control) or cells transfected with scramble siRNA (siScr) or TfR1-targeting siRNAs (siTfR1-1, siTfR1-2) at 72 h post-transfection. **h** Quantitative analysis of western blot for TfR1 expression shown in g. Data are presented as % of TfR1 expression in control cells (Control). Values represent mean ± SEM from n = 3 independent replicates. **i** Flow cytometry of viability of THP-1 macrophages treated as in g; data (% of live cells) are mean ± SEM from n = 3 independent replicates. **j and k** Flow cytometry of internalized Tfn-AF488 (25 µg/ml) (j) or HFt-AF488 (100 µg/ml) (k) by THP-1 macrophages after TfR1 knockdown within 30 min at 37 °C; untreated cells (Control) and siScr-transfected cells were controls. Data (% of ligand uptake in control cells) are mean ± SEM from n = 3 (Tfn-AF488) or n = 3 (HFt-AF488) independent replicates. Statistical analyses: two-way ANOVA with Sidak's post hoc test in d, f; one-way ANOVA with Dunnett's post-hoc test in b, h, j, k. ns P > 0.05, *P ≤ 0.05, **P ≤ 0.01, ***P ≤ 0.001, ***P ≤ 0.0001. Source data are provided as a Source Data file.

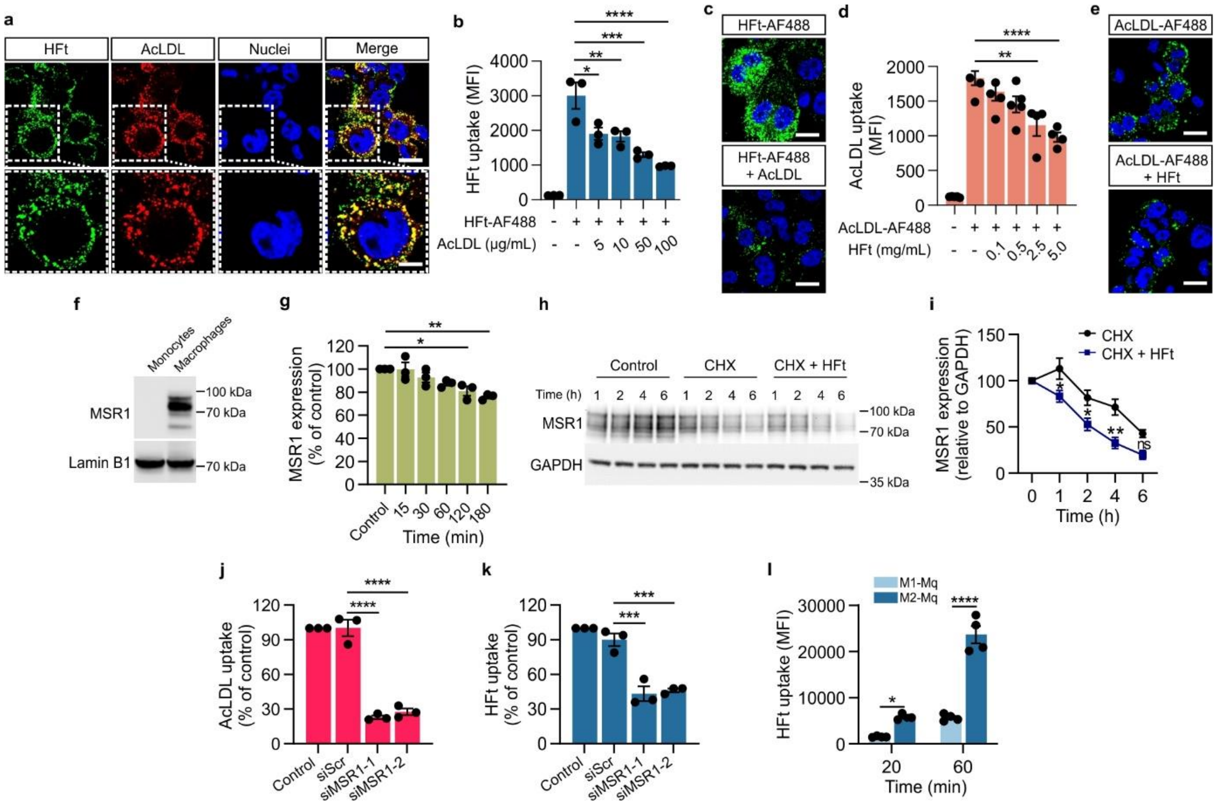

Supplementary Figure 6. MSR1 is involved in H-ferritin uptake by THP-1-derived macrophages. a Representative confocal microscopy images of HFt-AF488 and AcLDL-AF594 colocalization in THP-1 macrophages incubated with HFt-AF488 (50 µg/ml) and AcLDL-AF594 (5 µg/ml) for 15 min at 37 °C; nuclei stained with Hoechst 33342. Scale bar: 20 µm (10 µm in zommed region). **b** Flow cytometry of HFt-AF488 (5 µg/ml) uptake by THP-1 macrophages with or without AcLDL for 30 min at 37 °C; data shown as mean ± SEM from n = 3 independent replicates. **c** Representative confocal microscopy images of HFt-AF488 uptake by THP-1 macrophages with or without AcLDL (100 µg/ml) for 30 min at 37 °C; nuclei stained with Hoechst 33342. Scale bar: 20 µm. **d** Flow cytometry of AcLDL-AF488 (1 µg/ml) uptake by THP-1 macrophages with or without HFt for 30 min at 37 °C; data shown as mean ± SEM from n = 4 independent replicates. **e** Representative confocal microscopy images of AcLDL-AF488 uptake by THP-1 macrophages with or without HFt (2.5 mg/ml) for 30 min at 37 °C; nuclei stained with Hoechst 33342. Scale bar: 20 µm. **f** Western blot of MSR1 expression in THP-1 monocytes and macrophages. **g** Flow cytometry of MSR1 surface expression in THP-1 macrophages stimulated with HFt (200 µg/ml) for indicated times at 37 °C; untreated cells (Control) for comparison; data shown as mean ± SEM from n = 3 independent replicates. **h** Western blot of MSR1 expression in THP-1 macrophages untreated (Control), treated with cycloheximide (CHX, 20 µg/ml) for 1 h prior to HFt stimulation (200 µg/ml) (CHX+HFt) for indicated times at 37 °C; CHX-only treated cells used for comparison. **i** Quantitative analysis of relative MSR1 expression in THP-1 macrophages. **j–k** Flow cytometry of AcLDL-AF488 (5 µg/ml) (**j**) or HFt-AF488 (100 µg/ml) (**k**) uptake by THP-1 macrophages after MSR1 knockdown for 30 min at 37 °C; untreated cells (Control) and siScr-transfected cells used for comparison; data shown as mean ± SEM from n = 3 independent replicates. **l** Flow cytometry of HFt-AF488 uptake by M1 and M2 hMDM at 100 µg/ml for 20 or 60 min at 37 °C; data shown as mean ± SEM from n = 4 experiments. Statistical analysis: two-way ANOVA with Sidak's post hoc test in i and l; one-way ANOVA with Dunnett's test in b, d, g, j–k. For all panels, *P ≤ 0.05, **P ≤ 0.01, ***P ≤ 0.001, ****P ≤ 0.0001. Uncropped blots and source data are provided as a Source Data file.

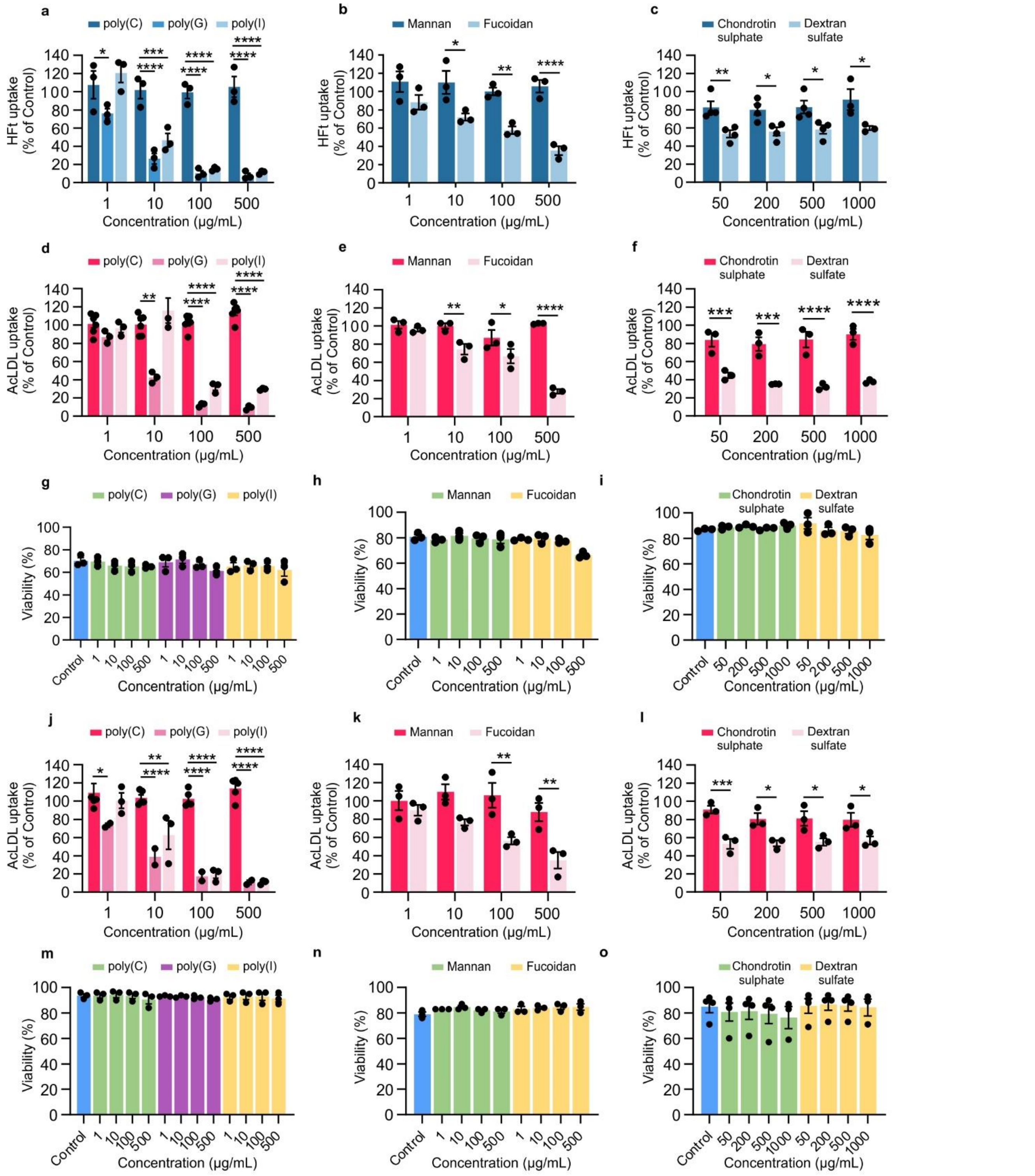

Supplementary Figure 7. Involvement of a scavenger receptor class A in H-ferritin uptake by human macrophages. **a-c** Flow cytometry of HFt-AF488 (50 µg/ml) uptake within 30 min at 37 °C by THP-1 macrophages, untreated or pre-treated for 30 min with class A scavenger receptor ligands or structurally related ligands that do not bind to this group of receptors (negative controls): poly(I), poly(G) and poly(C) (control) (a), fucoidan and mannan (control) (b) and dextran sulphate or chondroitin sulphate (control) (c) Flow cytometry data are presented as % of HFt-AF488 uptake in untreated, control cells. Values represent mean ± SEM from at least n = 3 independent replicates. **d–f**, **j–l** Flow cytometry of AcLDL-AF488 (5 µg/ml) uptake within 30 min at 37 °C by hMDM (**d–f**) or THP-1 macrophages (**j–l**), untreated or pre-treated as above with ligands or controls: poly(I), poly(G), poly(C) (**d**, **j**); fucoidan, mannan (**e**, **k**); dextran sulphate, chondroitin sulphate (**f**, **l**). Data are % of AcLDL-AF488 uptake in untreated control cells; mean ± SEM from n = 3 donors (hMDM) or n = 3 independent replicates (THP-1). **g–i**, **m–o** Flow cytometry of hMDM (**g–i**) or THP-1 macrophage (**m–o**) viability after 1 h treatment with ligands or controls as above. Untreated control cells were used for comparison. Data are % of live cells; mean ± SEM from n = 3 donors (hMDM) or n = 3 independent replicates (THP-

1). Statistical analysis: two-way ANOVA with Tukey's post-hoc test in panels **a–f**, **j–l**. For all panels, *P ≤ 0.05, **P ≤ 0.01, ***P ≤ 0.001, ****P ≤ 0.0001. Source data are provided as a Source Data file.

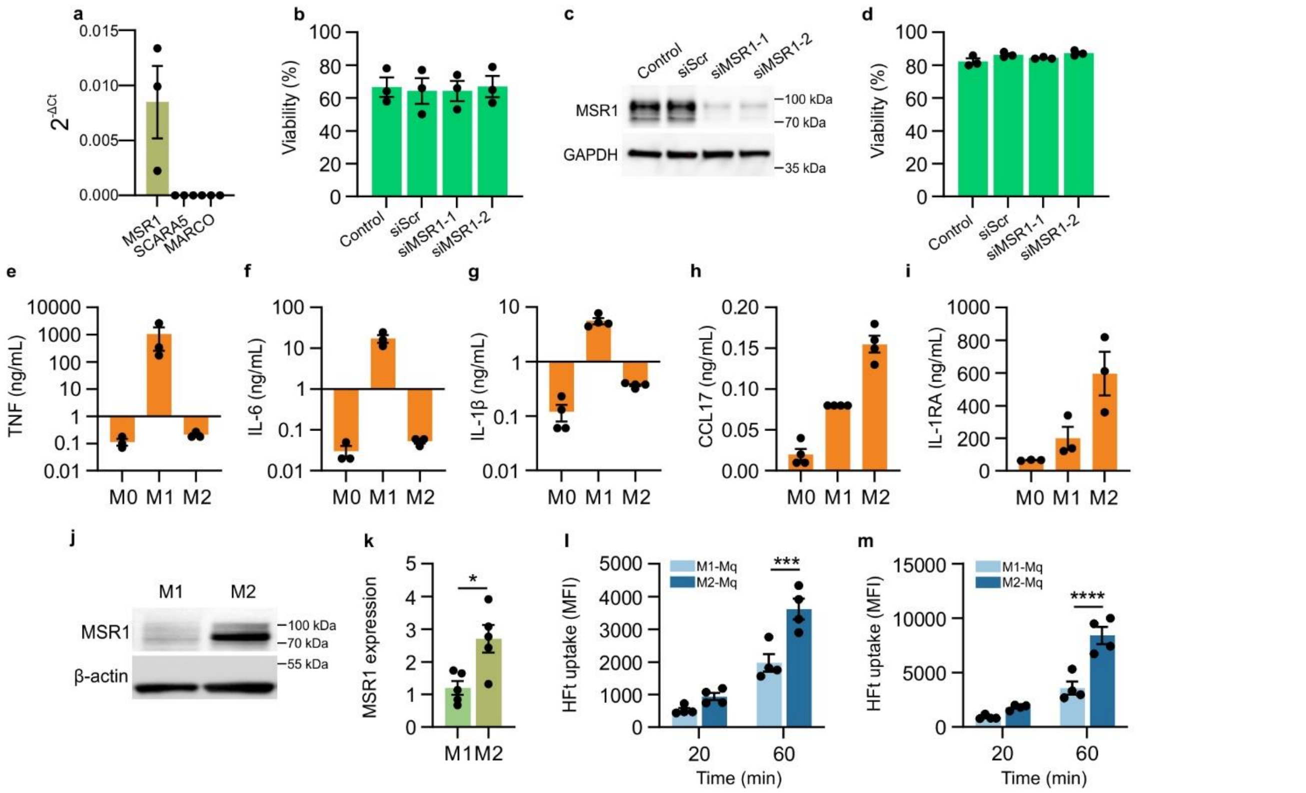

Supplementary Figure 8. MSR1-dependent uptake of H-ferritin in human macrophages. a Quantitative real-time PCR analysis of MSR1, SCARA5 and MARCO mRNA expression in THP-1 macrophages. **b** Flow cytometry analysis of hMDM viability at 72 h after MSR1 gene knock down with one of the following siRNA: no. 1 siRNA targeting MSR1 (siMSR1-1) or no. 2 siRNA targeting MSR1 (siMSR1-2). For comparison, untreated cells (Control) or cells electroporated with a negative, scramble siRNA (siScr) were used. Data is presented as % of live cells. Values represent mean ± SEM from n = 3 independent donors. **c** Western blot analysis showing MSR1 expression in either untreated THP-1 macrophages (Control) or cells transfected with one of the following siRNA sequences: scramble siRNA (siScr), no. 1 siRNA targeting MSR1 (siMSR1-1) or no. 2 siRNA targeting MSR1 (siMSR1-2) at 72 h after transfection. Representative western blot images are shown. **d** Flow cytometry analysis of THP-1 macrophages' viability treated as described in c. Data is presented as % of live cells. Values represent mean ± SEM from n = 3 independent replicates. **e-i** Bead-based immunoassay analysis of cytokines secreted by M0, M1 and M2 THP-1 macrophages. **j** Western blot analysis of MSR1 protein expression in THP-1 macrophages polarized to M1 or M2 phenotypes. Uncropped blots in Source Data. **k** Relative expression of MSR1 in M1 and M2 THP-1 macrophages quantified based on western blot analysis shown in j. **l** and **m** Flow cytometry analysis of internalized HFt-AF488 by M1 and M2 THP-1 macrophages given at 25 µg/ml (l) or 100 µg/ml (m) within 20 or 60 min at 37 °C. For comparison, untreated cells were used. Flow cytometry data are presented as mean fluorescence intensity (MFI) of HFt-AF488. Values represent mean ± SEM from n = 4 independent replicates. The Student's t-test was used for statistical analysis in a panels **k.** The two-way ANOVA followed by Sidak's multiple comparisons post hoc test were used for statistical analysis in panels l and m. For all panels, *P ≤ 0.05, ***P ≤ 0.001, ****P ≤ 0.0001. Source data are provided as a Source Data file.

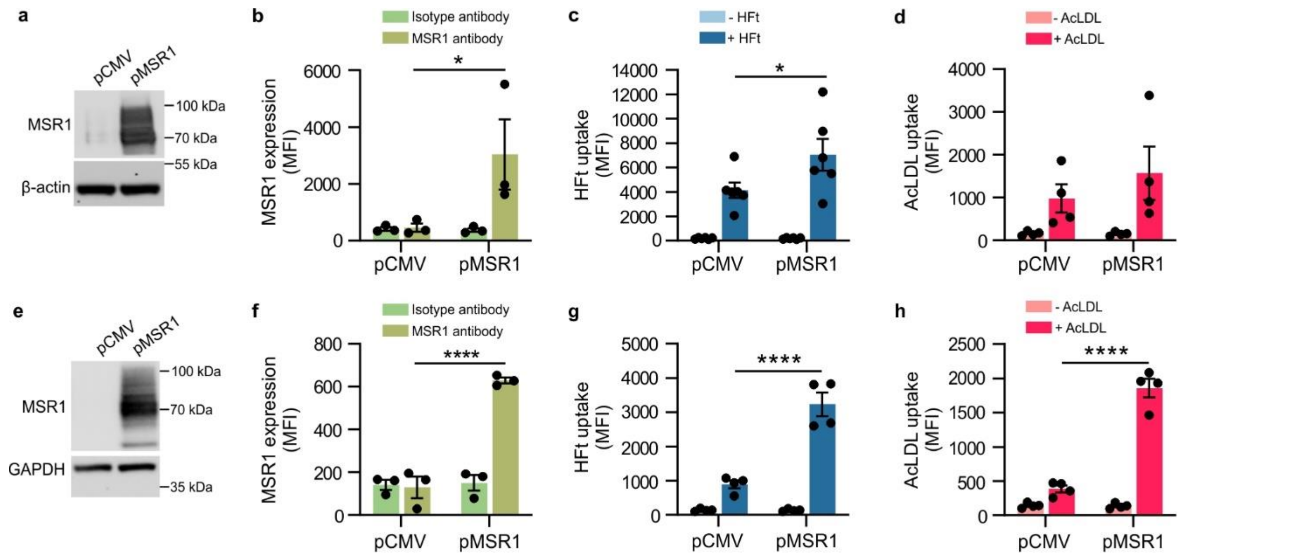

Supplementary Figure 9. The effect of MSR1 overexpression on H-ferritin uptake in HEK-293 and CHO-K1 cells. a, e Western blot analysis of MSR1 protein expression in HEK293 (a) and CHO-K1 (e) cells at 24 h after transfection with the control plasmid (pCMV) or the plasmid encoding the MSR1 gene (pMSR1). Representative western blot images are shown. **b, f** Flow cytometry analysis of cell surface expression of MSR1 in HEK293 (b) and CHO-K1 (f) cells at 24 h after transfection with the control plasmid (pCMV) or the plasmid encoding the MSR1 gene (pMSR1). Flow cytometry data are presented as mean fluorescence intensity (MFI) of PE. Values represent mean ± SEM from n = 3 independent replicates. **c, d** Flow cytometry analysis of internalized HFt-AF488 (100 µg/ml) (c) and AcLDL-AF488 (5 µg/ml) (d) within 30 min at 37 °C by HEK293 cells, transfected with either pCMV or pMSR1. Flow cytometry data is presented as mean fluorescence intensity (MFI) of fluorescently labeled ligands. Values represent mean ± SEM from at least four different replicates. **g, h** Flow cytometry analysis of internalized HFt-AF488 (100 µg/ml) (g) and AcLDL-AF488 (5 µg/ml) (h) within 30 min at 37 °C by CHO-K1 cells, transfected with either pCMV or pMSR1. Flow cytometry data is presented as mean fluorescence intensity (MFI) of fluorescently labeled ligands. Values represent mean ± SEM from four different replicates. The two-way ANOVA followed by Sidak's multiple comparisons post hoc test were used for statistical analysis. For all panels, *P ≤ 0.05, ****P ≤ 0.0001. Uncropped blots and source data are provided as a Source Data file.

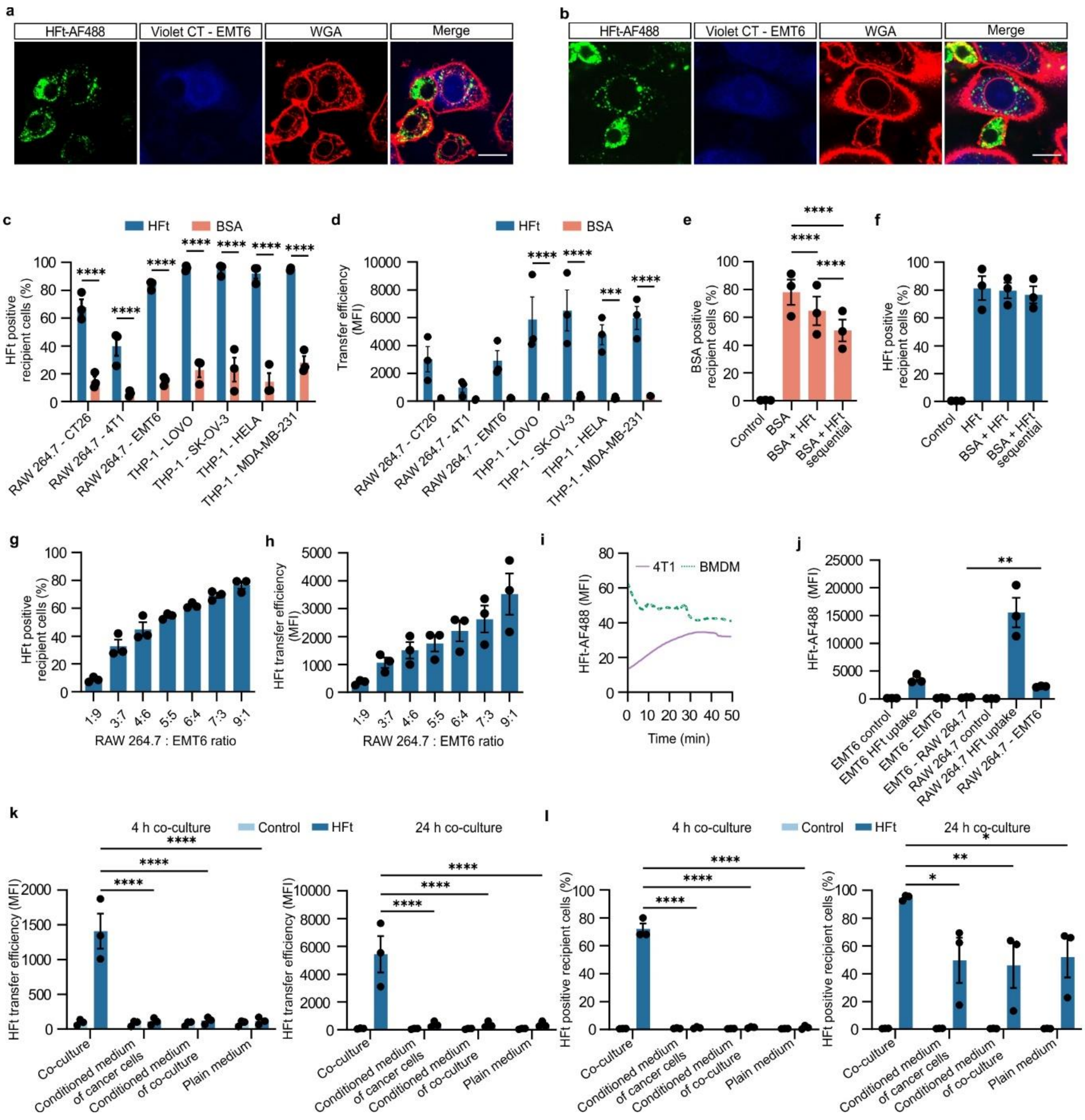

Supplementary Figure 10. Impact of various factors on HFt transfer from macrophages to surrounding cancer cells. **a-b** Representative confocal microscopy images of HFt-AF488 transfer from BMDM (a) and RAW 264.7 (b) (loaded with 500 µg/ml HFt-AF488 for 1 h at 37 °C) to EMT6 cells after 24 h co-culture. Scale bar: 10 µm. **c-d** Flow cytometry of HFt-AF488 and BSA-AF488 transfer from RAW 264.7 or THP1-derived macrophages (loaded with 1.5 µM conjugate for 1 h at 37 °C) to cancer cells after 24 h co-culture. Data is mean ± SEM; n = 3 independent replicates. **e-f** Comparison of (**e**) BSA-AF647 and (**f**) HFt-AF488 transfer from hMDM to MDA-MB-231 cells after 24 h co-culture. Macrophages were loaded with BSA, HFt, both (BSA + HFt), or sequentially with BSA then HFt (1.5 µM for 1 h at 37 °C). Empty hMDM served as control. Data is mean ± SEM; n = 3 independent replicates. **g-h** Flow cytometry of HFt-AF488 transfer from RAW 264.7 macrophages (loaded with 500 µg/ml HFt-AF488 for 1 h at 37 °C) to EMT6 cells co-cultured at varying ratios for 4 h; data are % of HFt-AF488-positive EMT6 cells (g) or MFI (h) ± SEM; n = 3 independent replicates. **i** HFt-FITC transfer from BMDM (loaded with 100 µg/ml HFt-FITC for 1 h at 37 °C) to 4T1 in co-culture. Mean FITC fluorescence intensity was quantified in cells from two separate time-lapse confocal microscopy imagings. **j** Flow cytometry of HFt-AF488 transfer: from EMT6 cells (loaded with 500 µg/ml HFt-AF488 for 1 h at 37 °C) to EMT6 cells or RAW 264.7 macrophages; and from RAW 264.7 macrophages (loaded as above) to EMT6 cells. Data is mean ± SEM; n = 3 independent replicates. **k-l** Flow cytometry of AF488 fluorescence in MDA-MB-231 cells after 4 and 24 h of co-culture with THP-1 macrophages loaded with HFt-AF488 or with conditioned media (CM) from THP-1-HFt-AF488 cultured 24 h in plain medium, cancer cell CM, or co-culture CM. Macrophages without ferritin were controls. Data is (k) geometric MFI of AF488 in cancer cells and (l) % of AF488-positive cancer cells ± SEM; n = 3 independent replicates. One-way ANOVA and Tukey HSD post-hoc test were used. For all panels, *P ≤ 0.05, **P ≤ 0.01, ****P ≤ 0.0001. Source data are provided as a Source Data file.

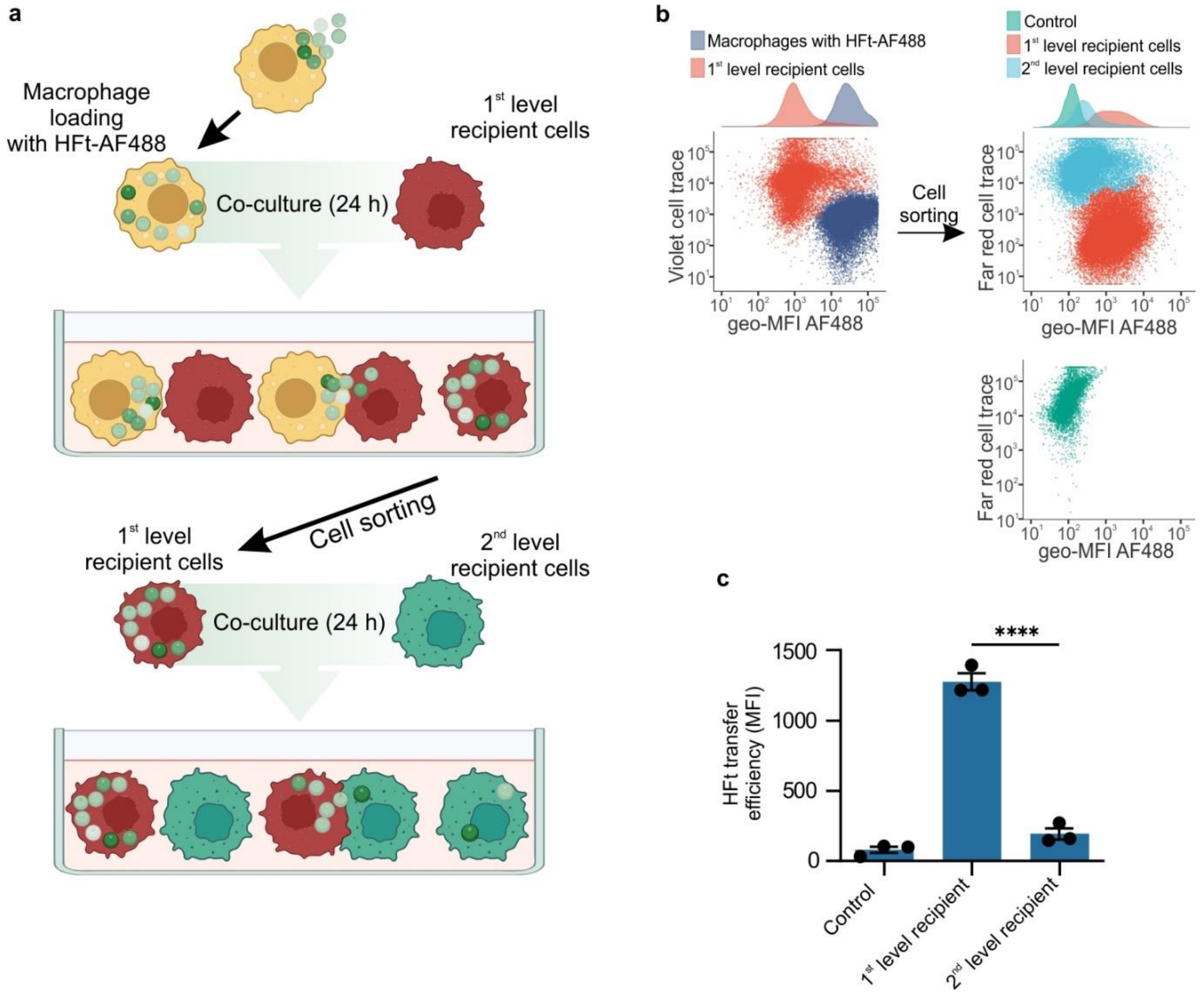

Supplementary Figure 11. Secondary HFt transfer between cancer cells in co-culture. a Schematic presentation of the experiment assessing secondary HFt transfer events between macrophages and cancer cells in co-culture. RAW264.7 macrophages loaded with HFt were co-cultured 24 h with recipient cancer cells (1st level), then separated using cell sorter and these cells were used as donor cells in subsequent co-culture with EMT6 as recipient cells (2nd level). Created in BioRender. Taciak, B. (2024) https://BioRender.com/p07x022. **b** Flow cytometry analysis of HFt-AF488 fluorescence in donor and recipient cells following 24 h co-culture compared to autofluorescence of corresponding controls. **c** Transfer efficiency from to the 1st level of recipient and to the 2nd level of recipient cells. Mean ± SEM of geo-MFI values of n = 3 independent replicates are presented on bar plot. Source data are provided as a Source Data file.

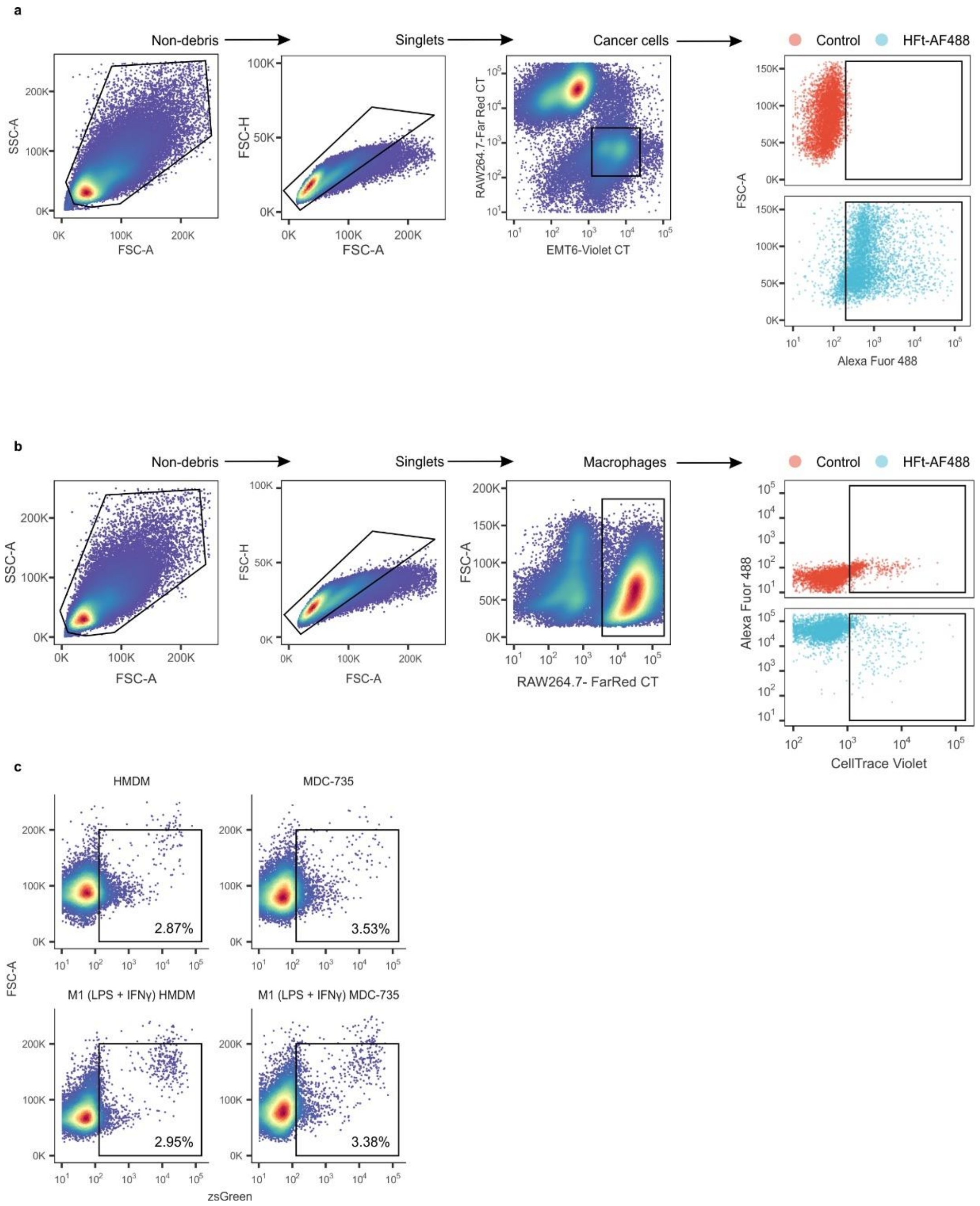

Supplementary Figure 12. HFt transfer is not phagocytosis-dependent. Representative flow cytometry scatterplots of macrophage-cancer cell co-cultures demonstrating HFt transfer without phagocytosis. **a** Scatterplots showing co-cultured macrophages labeled with HFt-AF488 and CellTrace Far Red, and cancer cells labeled with CellTrace Violet. Gating strategy separates CT Violet-positive cancer cells for analysis of HFt transfer. **b** Gating of macrophages in co-culture showing no significant increase in CT Violet-positive macrophages, indicating lack of cancer cell phagocytosis. **c** Representative scatterplots of co-cultures with MDC-735 and SK-OV-3-zsGreen cancer cells after 24 h, showing no induction of phagocytosis by drug-conjugate loading, even with LPS + IFNγ stimulation.

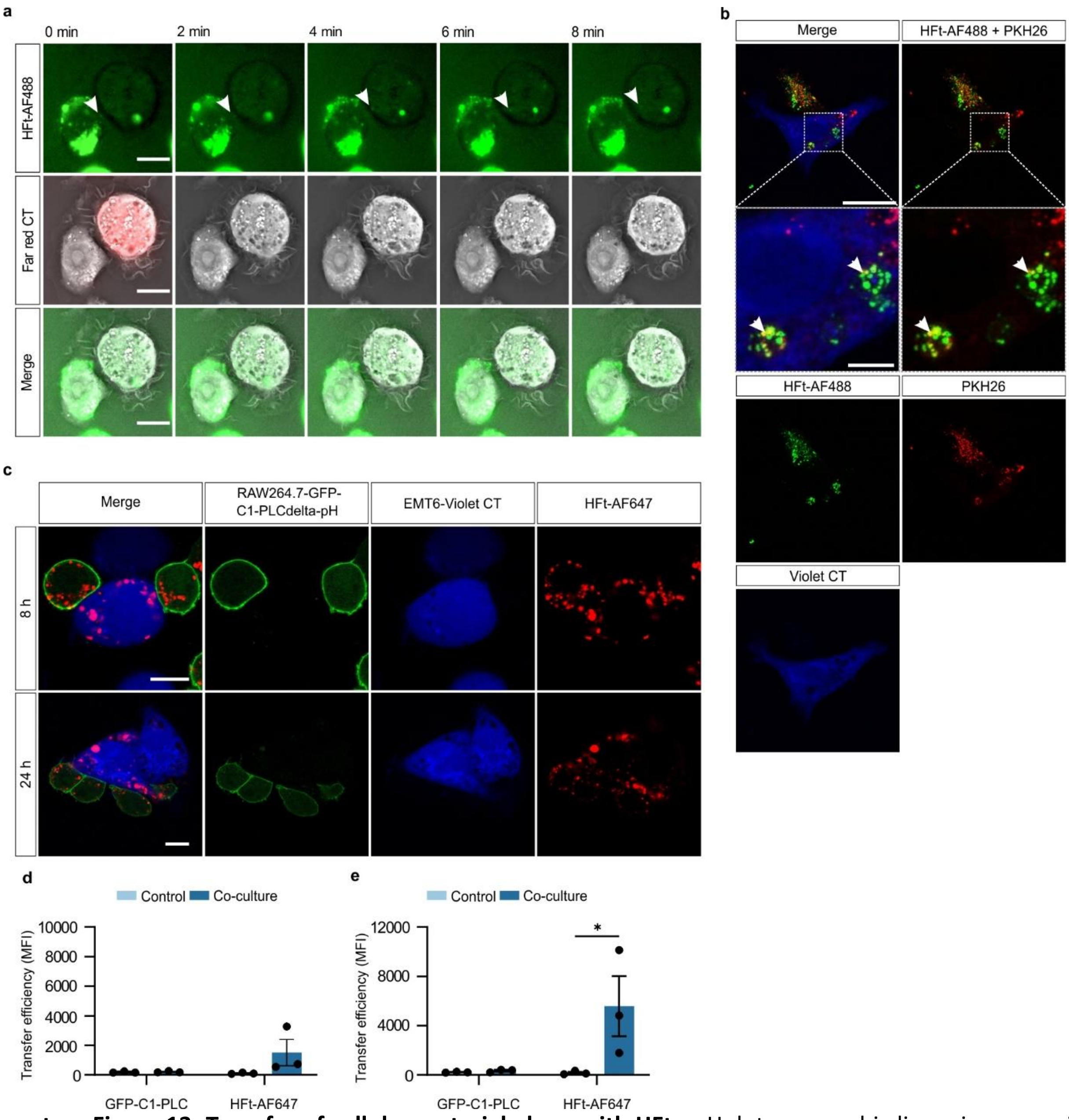

Supplementary Figure 13. Transfer of cellular material along with HFt. a Holotomographic live microscopy imaging of Ft transfer in a co-culture. Label free, refractive index (RI, gray) images of RAW264.7 macrophages loaded with HFt-Alexa Fluor 488 and EMT6 cancer cell (labeled with CellTrace Far Red) captured at several time-points. Arrowhead points green fluorescence spot exchanged between cells. Scale bar, 10 µm. **b** Representative confocal microscopy images captured after 24 h co-culture of donor hMDM labeled with PKH26 dye and loaded with HFt-AF488, and MDA-MB-231 cancer cells labeled with CellTrace Violet dye. Co-localization of AF488, and PKH26 signals both in macrophages and in cancer cells are pointed out by the arrowheads. Scale bar, 20 µm (5 µm in zoomed region). **c** Representative confocal microscopy images of RAW264.7 macrophages expressing GFP in the plasma membrane, loaded with HFt-AF647 co-cultured 8 and 24 h with EMT6 cancer cells stained with Violet CT cytoplasmic dye. Scale bar, 10 µm. **d-e** Flow cytometry analysis of GFP and AF647 fluorescence in EMT6 cells after **c** 4 and **d** 24 h co-culture with RAW264.7 expressing GFP in plasma membrane and loaded with HFt-AF647. Data shown on bar plots are mean ± SEM values from n = 3 independent replicates. The one-way ANOVA and Tukey HSD post-hoc test were used for statistical analysis. For all panels, * P ≤ 0.05. Source data are provided as a Source Data file.

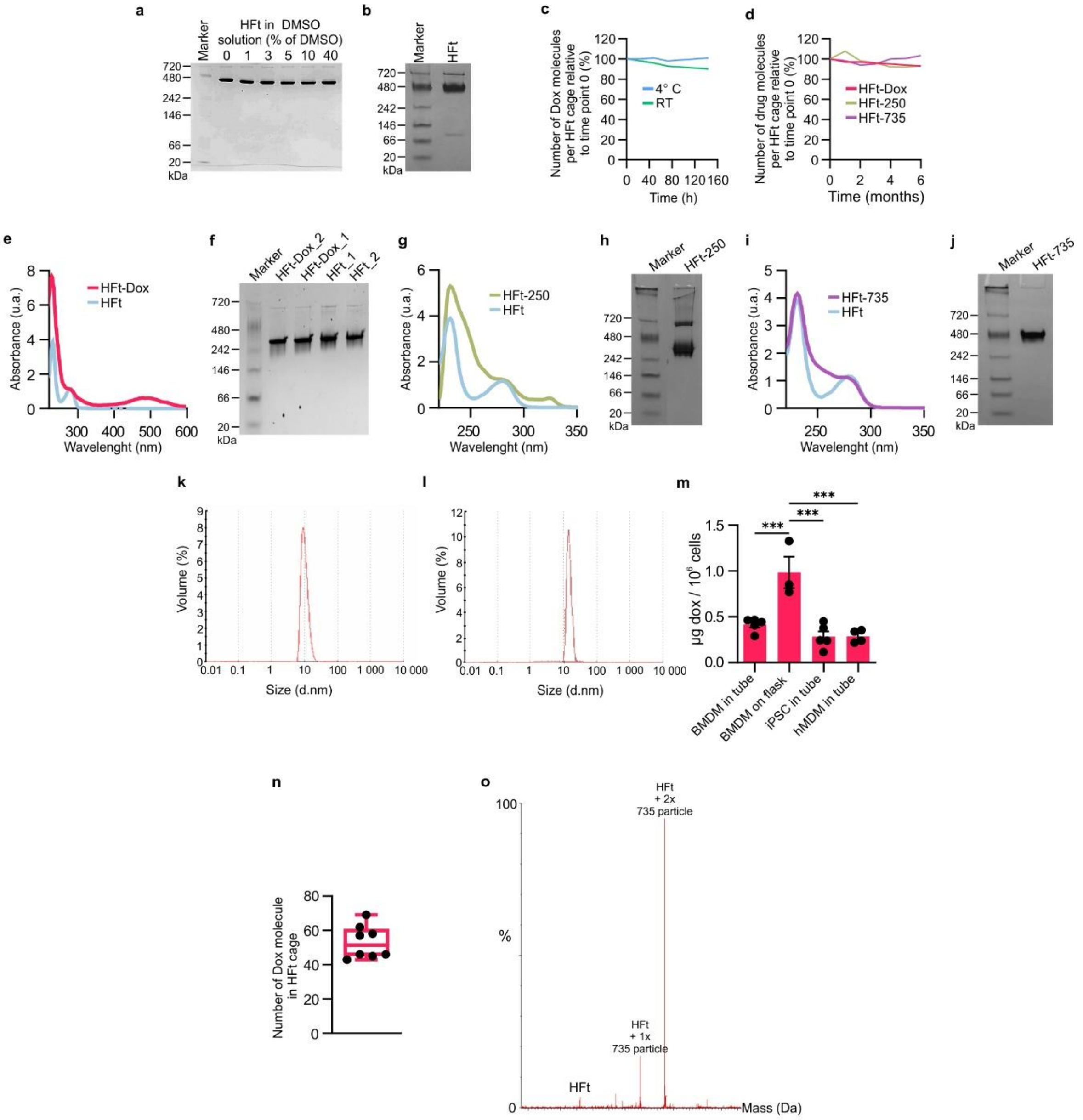

Supplementary Figure 14. Ferritin complexation with the drugs and stability of the complex. a Native PAGE analysis of HFt 24-mer in the presence of DMSO**. b** Native PAGE analysis of HFt. **c** Stability of HFt-Dox complex (drug molecules per HFt cage) at 4 °C and RT up to 160 h. **d** Stability of HFt-Dox, HFt-250, and HFt-735 complexes (drug molecules per HFt cage) at –80 °C up to 6 months. **e** UV-Vis spectra of 0.8 mg/ml of HFt with 46 molecules of doxorubicin per cage, calculated using the doxorubicin molar extinction coefficient of 10 420 M−1 cm−1 at 485 nm. The spectral difference between encapsulated and pure HFt at 480 nm indicates doxorubicin presence. **f** Native PAGE of two HFt 24-mer batches (batch 1 and 2) after doxorubicin encapsulation and unmodified control. **g** UV–Vis spectra of HFt and HFt-250 complex. Pure 250 drug shows absorbance maxima at 290 nm and 324 nm, visible in HFt-250 complex spectrum. Encapsulation efficiency calculated using drug's molar extinction coefficient at 324 nm ($\varepsilon = 7238\ M^{-1}\ cm^{-1}$) **h** Native PAGE of HFt-250 complex shows main band around 350 kDa, corresponding to 24-mer formation. **i** UV–Vis spectra of HFt and HFt-735 conjugate. Spectral difference at 252 nm indicates presence of 735 drug. **j** Native PAGE of HFt-735 conjugate shows main band around 400 kDa, corresponding to 24-mer formation and preservation of native form of HFt. **k** DLS results for HFt-250 complex: particles measured 11.76 nm diameter (100%), corresponding to 24-mers; complex homogeneity was 99%. **l** DLS of HFt-735 conjugate: hydrodynamic diameter measured 14.81 ± 2.70 nm; conjugate homogeneity was 99.9%. **m** Quantification of doxorubicin loaded into BMDM and hMDM, measured via doxorubicin fluorescence on TECAN plate reader. **n** Number of doxorubicin molecules loaded into HFt 24-mer after multiple loading cycles, presented as median. **o** Mass spectrometry of HFt-735 indicates fractions of double- and single-labeled conjugate and free protein. Uncropped blots and source data are provided as a Source Data file.

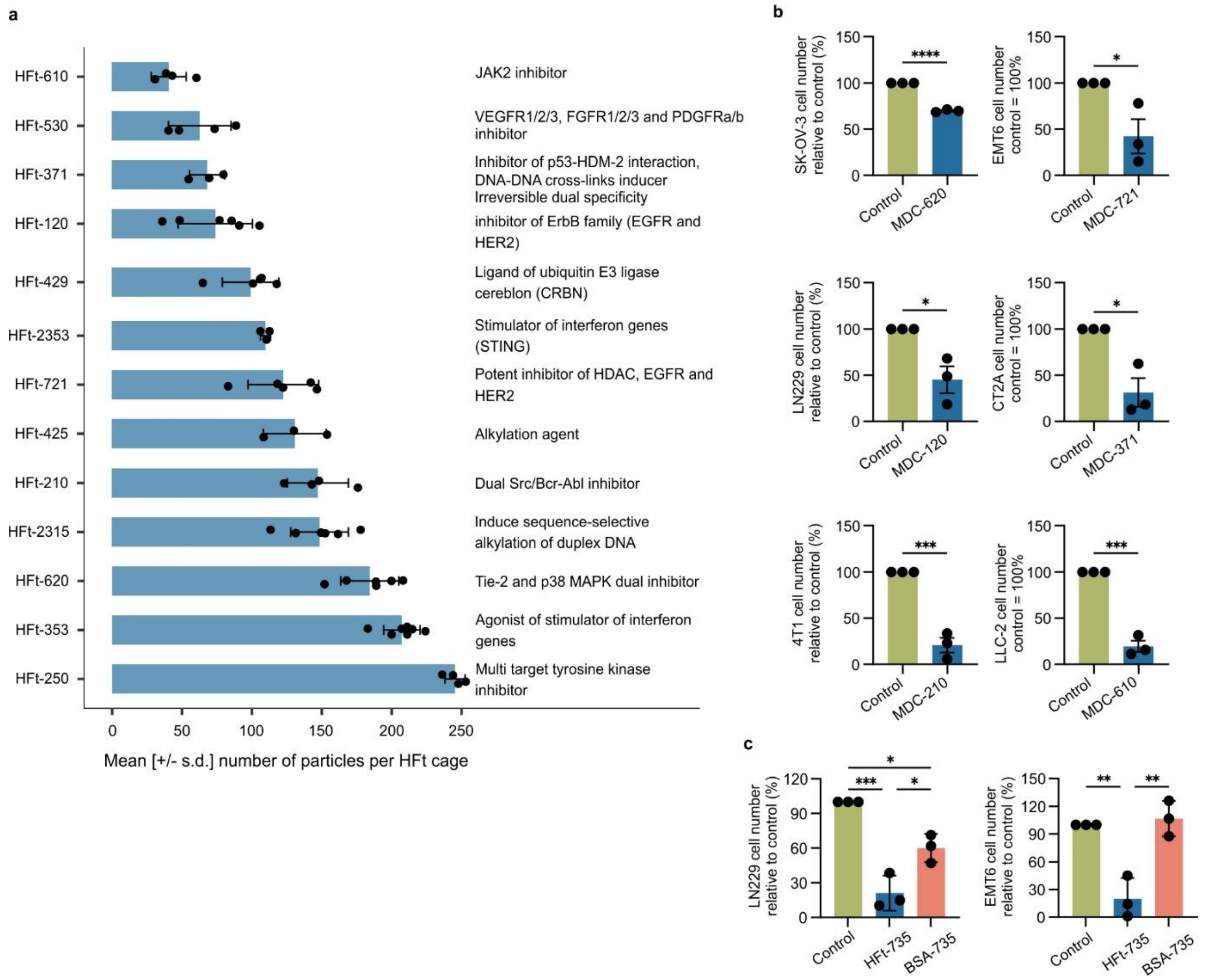

Supplementary Figure 15. Encapsulation efficiency of various drugs in HFt nanocages and efficacy *in vitro*. a Mean ± s.d. numbers of drug molecules encapsulated per HFt nanocage for various drug-HFt complexes, drug names are encoded. Adjacent to the bars, a brief description of the drug's mechanistic properties is provided. Each complexation experiment was performed between 1 to 7 times (n = 3-7). **b** In vitro evaluation of anticancer activity of macrophages loaded with selected HFt-drug complex or plain medium (Control) in co-culture with various cancer cell lines. T test was used for statistical analysis. Data is presented as mean ± s.d. of n = 3 independent replicates. The one-way ANOVA followed by Tukey post hoc test was used for statistical analysis; *P ≤ 0.05, **P ≤ 0.01, ***P ≤ 0.001, ****P ≤ 0.0001 **c** Cancer cell killing in 72 h co-culture of HMDM with LN229 glioma cells and BMDM with EMT6 breast cancer cells. Macrophages were incubated with BSA-735 or FT-735 at 0.1 mg/ml. Data are mean ± s.d. from n = 3 independent replicates. Source data are provided as a Source Data file.

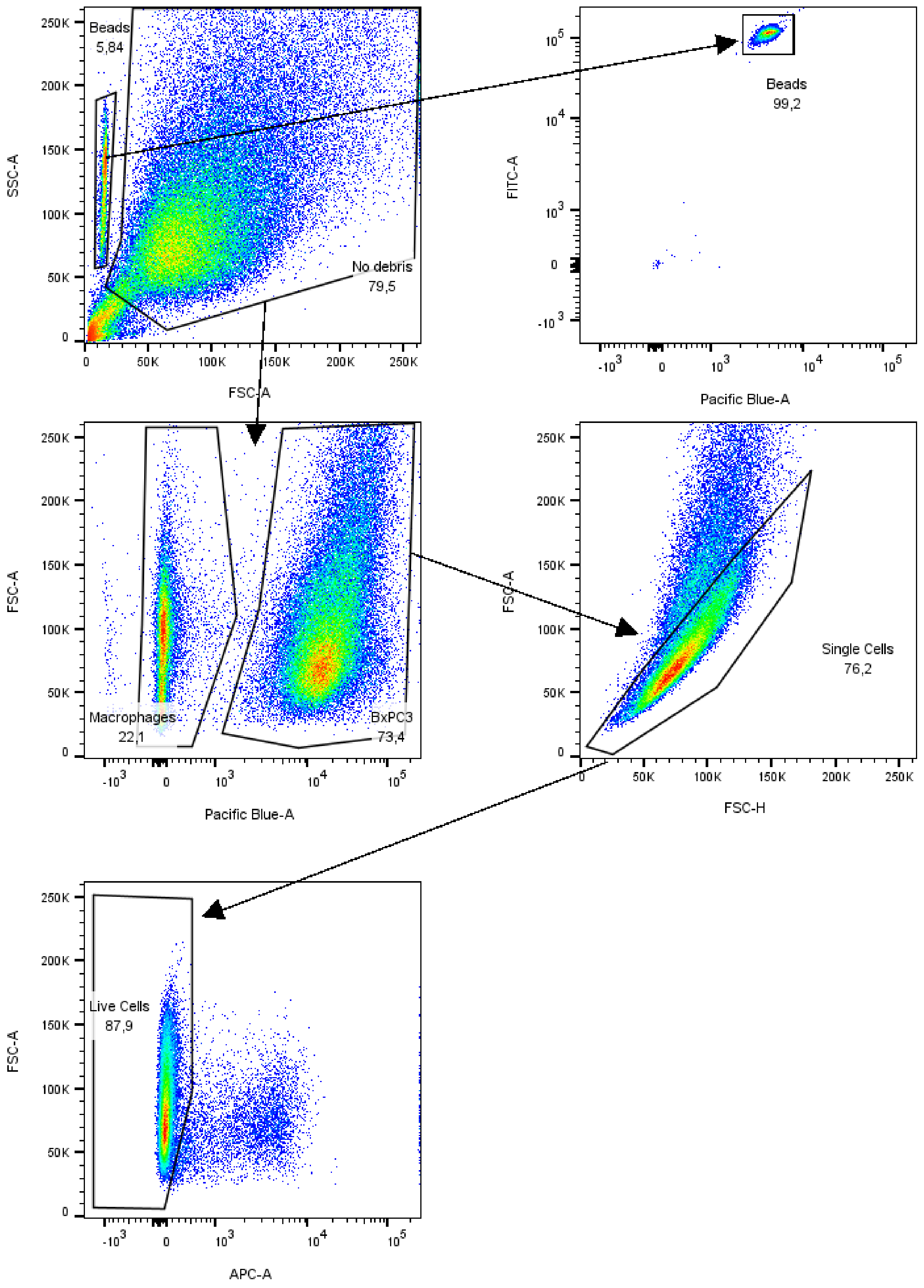

Supplementary Figure 16. Gating strategy for analysis of in vitro cytotoxicity co-culture assay. The gating starts with the use of forward scatter (FSC-A) and side scatter (SSC-A) to identify cell events and remove cell debris. Gating of CountBright Absolute Counting Beads was confirmed on two fluorescence channels. CellTrace dyes were used to distinguish macrophages from cancer cells. Forward scatter height (FSC-H) versus area (FSC-A) gating was used to select single cells. Live cells were then identified based on their lack of staining with a viability dye.

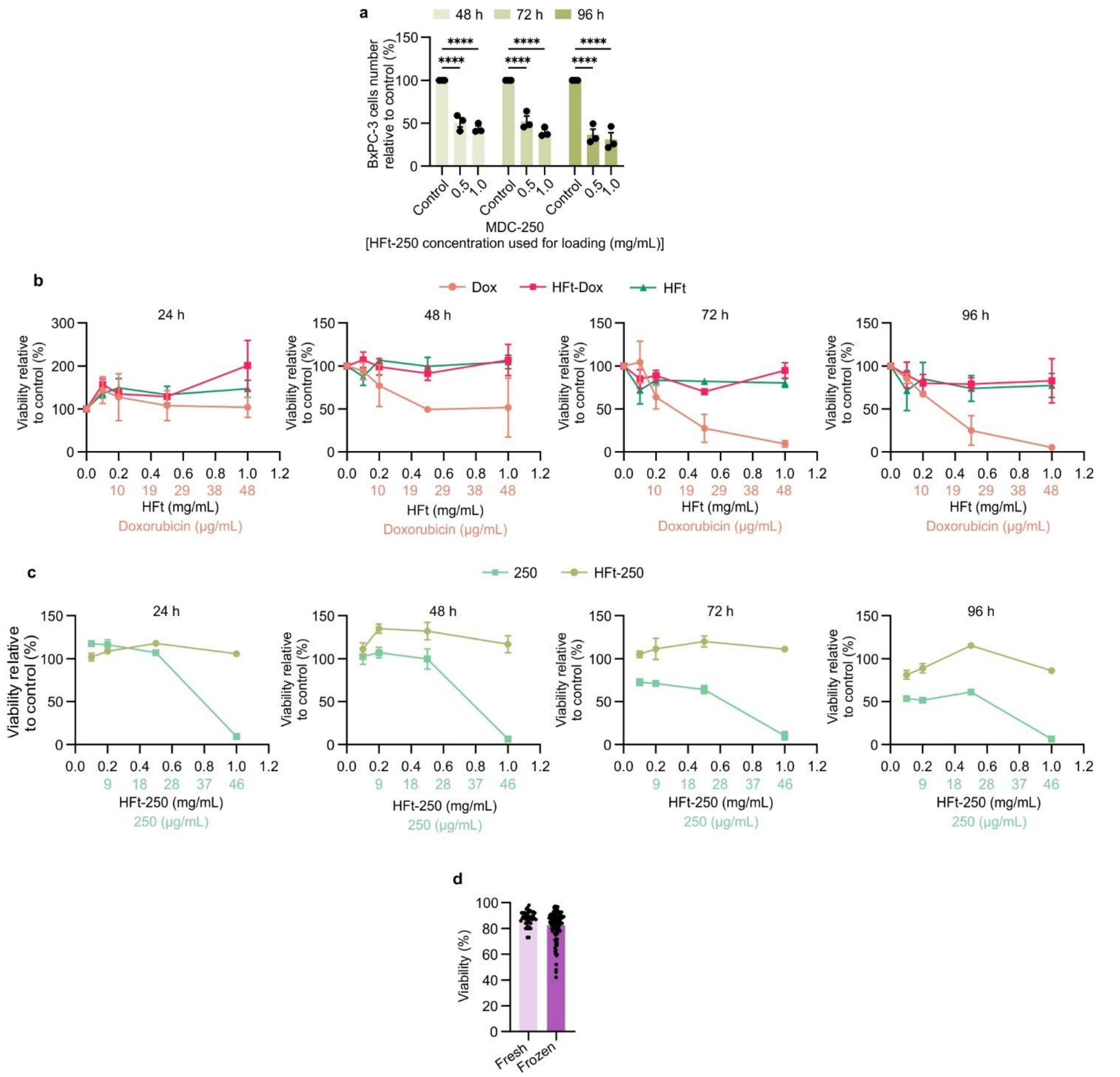

Supplementary Figure 17. Effect of HFt-drug conjugates on macrophages as MDC. a In vitro evaluation of cancer cell killing by human MDC against BxPC-3 pancreatic cancer cell line in co-culture. Macrophages were incubated with either 0.5 mg/ml or 1 mg/ml of HFt-250 complex or plain medium (Control). The one-way ANOVA and Tukey HSD post-hoc test was used for statistical analysis; ***P ≤ 0.001, ****P ≤ 0.0001. Data are presented as mean ± SEM of n = 3 independent replicates. **b** MTT analysis of hMDM viability at 24, 48, 72, and 96 h following loading with HFt-Dox, plain doxorubicin, and HFt, x axis shows HFt concentration; the concentration of plain doxorubicin used was equivalent to those encapsulated in HFt. **c** MTT analysis of hMDM viability at 24, 48, 72, and 96 h following loading with HFt-250 and plain 250 compound, x axis shows HFt concentration; the concentration of plain 250 compound used was equivalent to those encapsulated in HFt. **d** Viability of MDC-735 freshly loaded with HFt-735 drug compared with cryopreserved and thawed MDC-735. Data are presented as mean ± SEM from n = 43 (Fresh) or n = 158 (Frozen) samples. Statistical analysis was performed using a t-test. Source data are provided as a Source Data file.

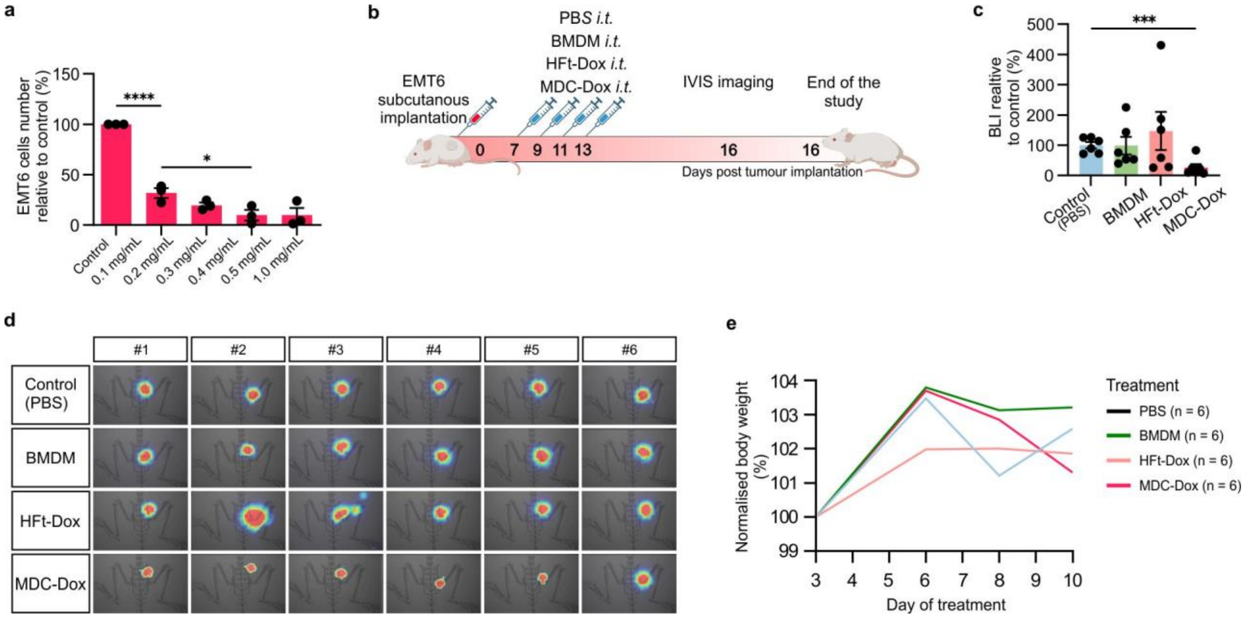

Supplementary Figure 18. Anticancer activity and therapeutic efficacy of MDC-Dox. a In vitro evaluation of EMT6 cancer cell killing by MDC-Dox in 96 h co-culture. BMDM macrophages were incubated with various HFt-Dox concentrations or plain medium (Control). Data presented is a mean ± SEM of n = 3 independent replicates. The one-way ANOVA and Tukey HSD post-hoc test were used for statistical analysis. **b** Schematic representation of the treatment schedule employed in the in vivo study: 1 × 10^6 of BMDMs loaded with HFt-doxorubicin (MDC-Dox), HFt-doxorubicin (HFt-Dox) given at the dose ensuring equivalent of doxorubicin administered in BMDMs, and plain BMDMs were administered i.t. Created in BioRender. Taciak, B. (2024) https://BioRender.com/y36i224. **c** In vivo analysis of mean bioluminescence intensity of EMT6-luc tumors after intratumoral treatment with MDC-Dox measured at the end of the experiment and **d** IVIS images of the tumors at the end of experiment in the control and MDC-Dox groups (n = 6 mice per group). Welch's t-test with Bonferroni multiple comparison correction was used for statistical analysis. **e** Mean body weights of mice during the course of the experiment of intratumoral treatment of EMT6-luc breast cancer with PBS, BMDM cells, HFt-Dox and MDC-Dox (n = 6 mice per group). For all panels, **P ≤ 0.01, ***P ≤ 0.001, ****P ≤ 0.0001. Source data are provided as a Source Data file.

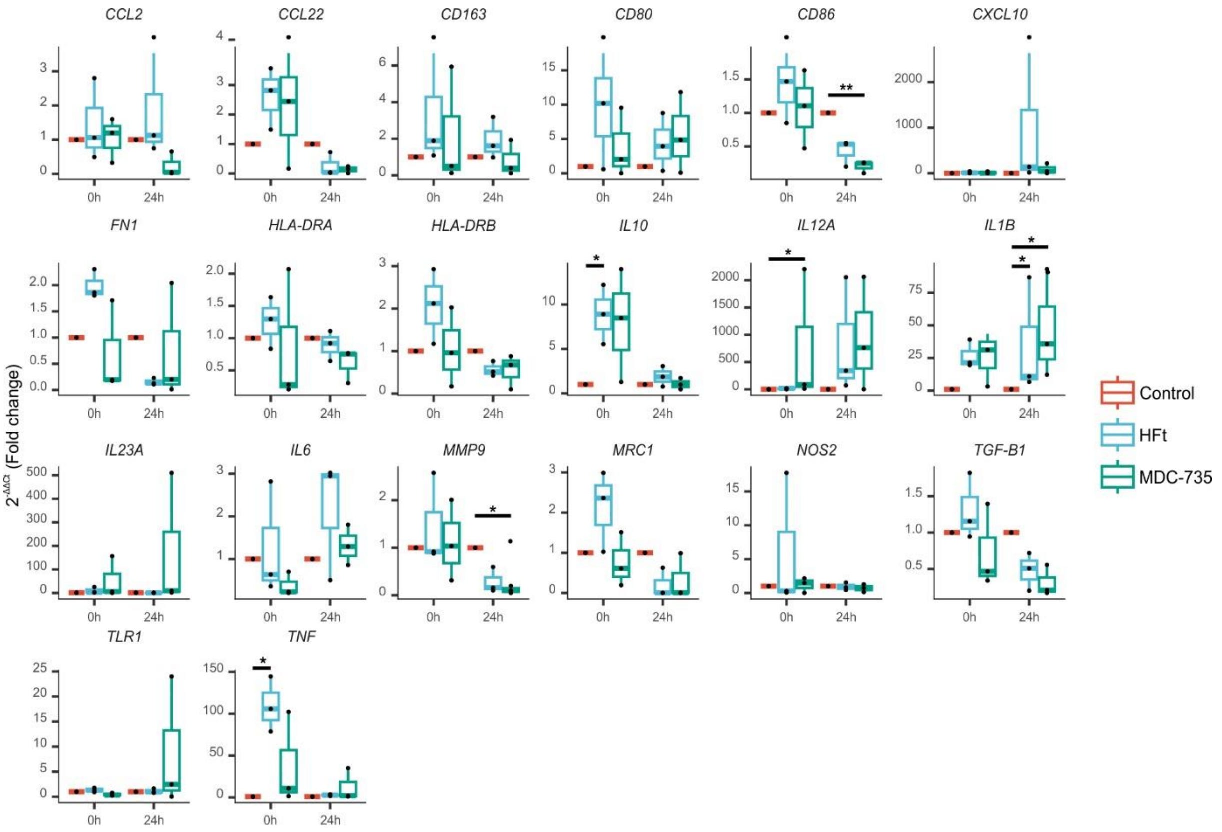

Supplementary Figure 19. Effect of HFt and HFt-735 loading on macrophages. RT-PCR analysis of selected macrophage marker gene expression in control hMDM and hMDM loaded with plain HFt or HFt-735 (at time-point 0h, directly following 1-hour incubation with ferritin) and after 24 h. Gene expression is shown as fold change, normalized to the ACTB housekeeping gene, and expressed relative to control macrophages not incubated with ferritin. n = 3 donors (hMDM source). Statistical analysis was performed using one-way ANOVA with Tukey's post-hoc test for multiple comparisons, *P ≤ 0.05, **P ≤ 0.01. Source data are provided as a Source Data file.

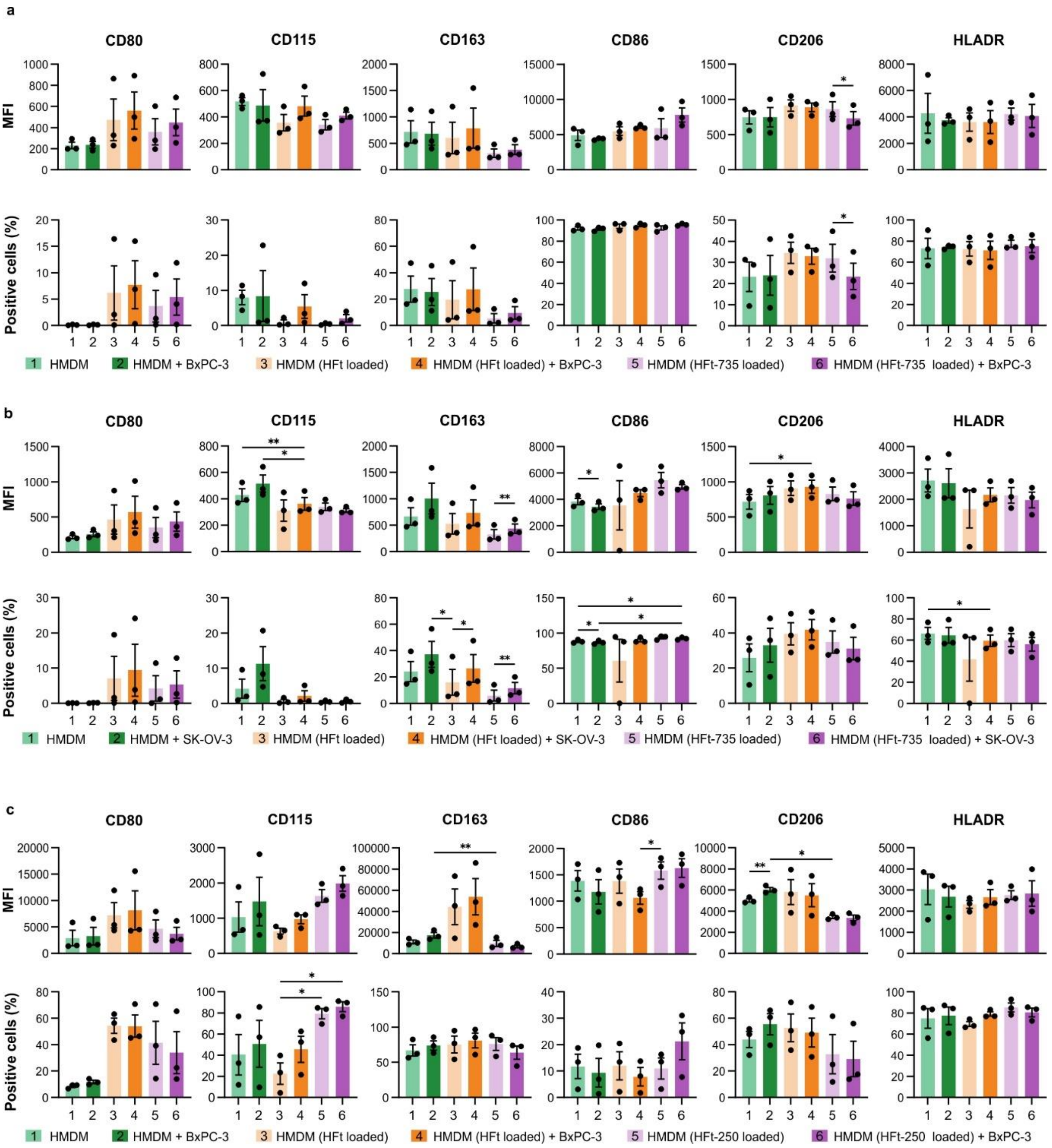

Supplementary Figure 20. Effect of HFt-drug loading and co-culture with cancer cells on macrophages. Flow cytometry analysis of changes in macrophage surface marker expression after treatment with PBS (hMDM) serving as a control, and hMDM loaded with free HFt (hMDM HFt loaded), or loaded with HFt-250 or HFt-735 drug conjugates. These macrophages were then co-cultured with either BxPC-3 **(a, c)** or SK-OV-3 **(b)** cancer cell lines. Panels **(a)** and **(b)** represent the effects of co-culturing HFt-735 loaded hMDM with BxPC-3 and SK-OV-3 cells, respectively, while panel **(c)** shows the effects of co-culturing HFt-250 loaded hMDM with BxPC-3 cancer cells. Data presented as MFI ± SEM from n = 3 independent replicates (hMDM obtained from 3 donors). The statistical analysis was conducted using one-way ANOVA test complemented by Tukey's post-test for multiple comparisons. For all panels, *P ≤ 0.05, **P ≤ 0.01. Source data are provided as a Source Data file.

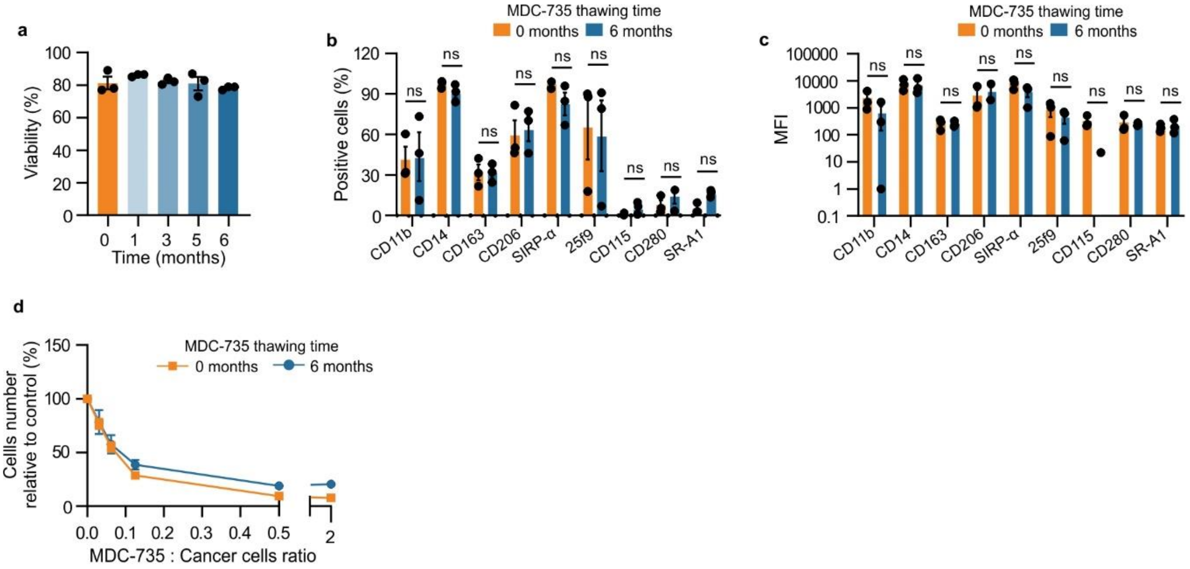

Supplementary Figure 21. Stability of the MDC-735 product. a Viability results of MDC-735 that was cryopreserved and thawed (time point 0 was a starting point) and 1, 3, 5 and 6 months later, n = hMDM from 3 donors. **b, c** Flow cytometry analysis of changes in macrophage surface marker expression at time point 0 and after 6 months, showed as % of positive cells (**b**) and MFI (**c**), n = hMDM from 3 donors. **d** Cytotoxic activity of MDC-735 (thawed at time point 0 and 6) against LN-229 cells in vitro. The results are presented as a percentage of live cancer cells relative to the number of live cancer cells in the control, n = 3 independent replicates (hMDM obtained from 3 donors). The statistical analysis of the data was conducted using one-way ANOVA and Tukey's post-hoc test for multiple comparisons. For all panels, ns = not significant. Source data are provided as a Source Data file.

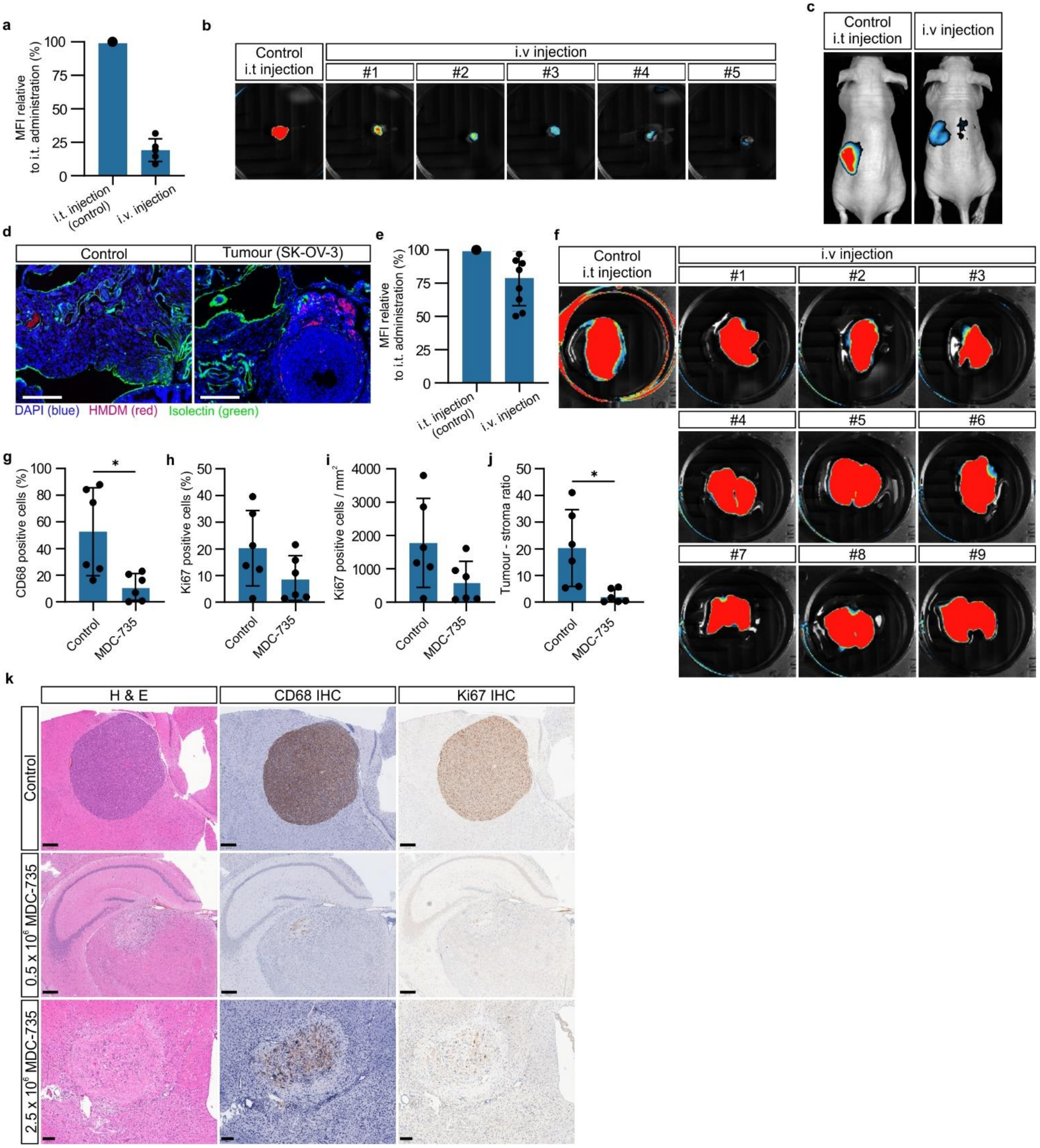

Supplementary Figure 22. MDC migrate to the tumor mass and interact with tumor stroma. a, e Mean Fluorescence Intensity (MFI) of hMDMs labeled with XenoLight DiR in target tumor tissue. 5 mln of labeled hMDM were i.v. injected to mice with orthotopic ovarian cancer SK-OV-3 (**a**) and cancer in the lung A549-luc and MDA-MB-231-luc (**e**). The graph shows an average MFI of tumor relative to control (i.t. administration of 5 mln of hMDMs labeled with XenoLight DiR). **b, c, f** Representative fluorescence images of tumor-bearing ovaries (**b**) dissected from the animals with orthotopic SK-OV-3 tumors(**c**); and pictures of lungs (**f**) dissected from animals with lung growing A549-luc or MDA-MB-231-luc tumors, following intravenous (*i.v.*) injection of hMDM-XenoLight DIR (red). Control animal received full dose of labeled hMDM directly to the tumor. **d** Mean Fluorescence Intensity (MFI) of hMDMs labeled with XenoLight DiR in Control (healthy) and SK-OV-3 tumor-bearing mice. Nuclei were counterstained with DAPI (blue), and vasculature was stained using Isolectin-IB4-AF488 (green). Scale bar = 200 µm. **a-f** n = 5-9 mice per group. **g** Percentage of the tumor area stained positive for CD68 (30-day samples). Data shown are mean ± SEMf from n = 6 mice per group. **h-i** Ki67 positive staining in U87-MG tumors 30 days after treatment with PBS or MDC-735. Data shown are mean ± SEMf from n = 6 mice per group. **j** Tumor – stroma ratio in U87-MG tumors 30 days after treatment with PBS or MDC-

735. **k** Representative brain sections from U87-MG tumor-bearing mice 30 days post-treatment with PBS (Control) or two different doses of MDC-735. Sections show H&E staining and immunohistochemistry for Ki67 and CD68. Scale bar = 250 µm (Control and MDC-735 0.5×10^6) or 100 µm (MDC-735 2.5×10^6). Source data are provided as a Source Data file.

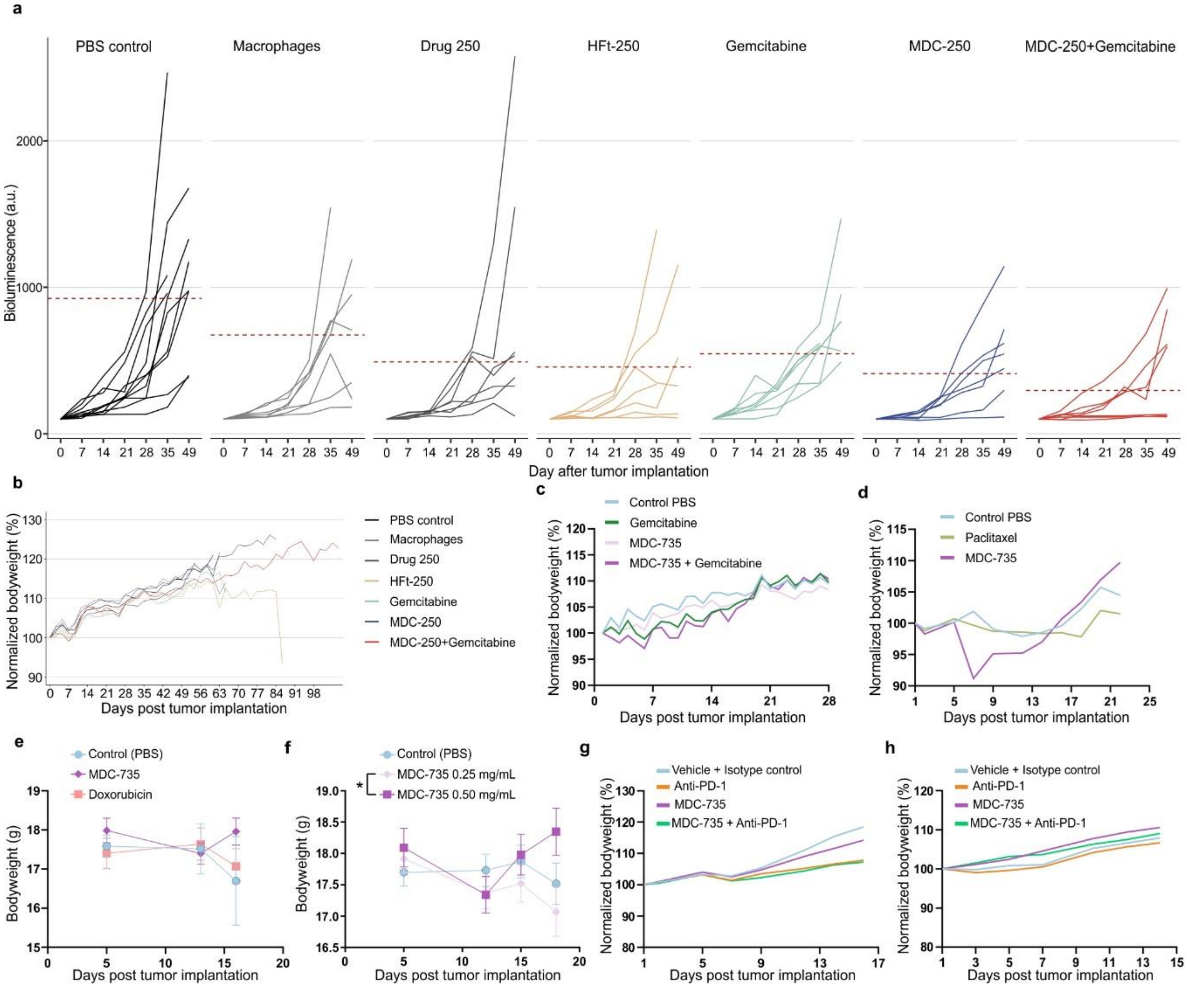

Supplementary Figure 23. MDC-735 therapy inhibits tumor growth and does not affect mouse weight. a Quantitative analysis of the bioluminescence signal in female athymic nude mice bearing orthotopic BxPC-3 tumors treated with MDC-250 and controls. The dashed line shows the mean signal value for each group on day 35 (the last day on which all animals were still alive). **b** Relative changes in normalized body weight of female athymic nude mice bearing orthotopic BxPC-3 tumors treated with MDC-250 and control groups. Values shown are mean; n = 7-10 per group. **c** Relative changes in normalized body weight of female athymic nude mice bearing orthotopic BxPC-3 tumors treated with MDC-735 and control groups. Values shown are mean; n = 8 for all groups. **d** Relative changes in normalized body weight of female athymic nude mice bearing orthotopic SK-OV-3 tumors treated with MDC-735 and control groups. Values shown are mean ± SEM; n = 8 for all groups. **e** Changes in body weight of female BALB/c mice with breast EMT6 cancer metastasis to the lungs treated with mouse MDC-735 and control groups. Values shown are mean ± SEM; n = 7-8 per group. **f** Changes in body weight of female BALB/c mice with breast EMT6 cancer metastasis to the lungs treated with mouse MDC-735 prepared by macrophage loading with two different FT-735 concentrations and a control group. Values shown are mean ± SEM; n = 15-20 per group. **g** Relative changes in normalized body weight of male C57BL/6 mice bearing MB49 bladder tumors in the flank. Values shown are mean; n = 10 for all groups. **h** Relative changes in normalized body weight of female C3H/HeN mice bearing SCC7 squamous cell carcinoma tumors in the flank. Values shown are mean; n = 9-10 per group. Source data are provided as a Source Data file.

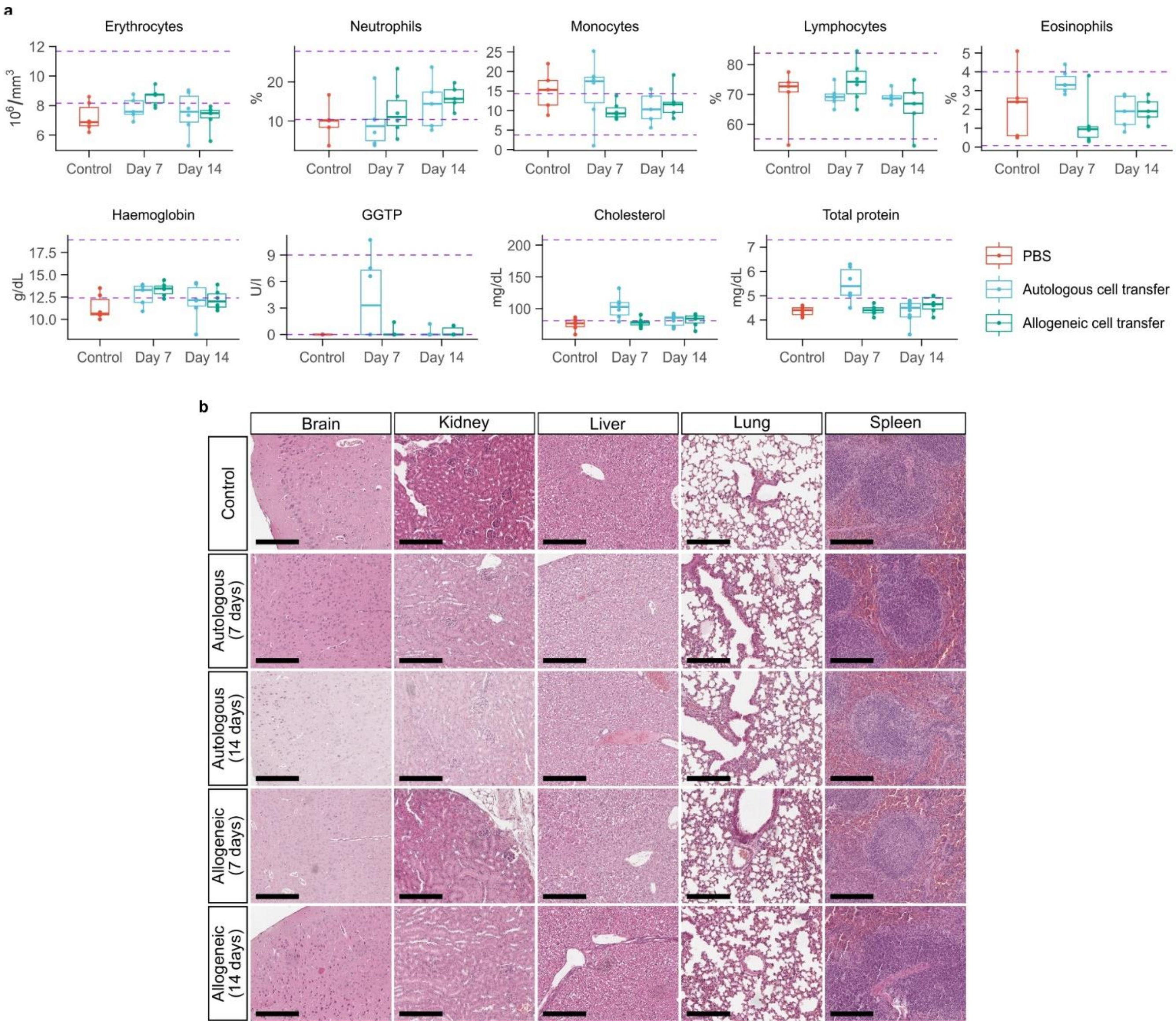

Supplementary Figure 24. In vivo safety evaluation of MDC therapy. a Blood analysis and **b** representative histopathology findings (brain, kidney, liver, lung and spleen) of Balb/c naïve mice received 15 × 10^6 of autologous BMDMs or 15 x 10^6 of allogeneic BMDMs (from C57BL/6 mice) or PBS (control) compared to the reference value (blood) and control mice (naive); n = 5 - 10 mice per group. (a) Box plots indicate median (middle line), 25th, 75th percentile (box) and 5th and 95th percentile (whiskers). (b) Scale bar = 200 µm. Source data are provided as a Source Data file.

Supplementary Tables

Supplementary Table 1. Primer sequences used for RT-PCR. This table lists the forward and reverse primer sequences for the target genes, along with β-actin (ACTB) as the housekeeping gene used for normalization.

Gene name	Entrez Gene ID	Forwad primer	Reverse primer
TNF	7124	GAGGCCAAGCCCTGGTATG	CGGGCCGATTGATCTCAGC
IL6	3569	CCTGAACCTTCCAAAGATGGC	TTCACCAGGCAAGTCTCCTCA
Il1B	3553	TTCGACACATGGGATAACGAGG	TTTTTGCTGTGAGTCCCGGAG
Il12A	3592	ATGGCCCTGTGCCTTAGTAGT	AGCTTTGCATTCATGGTCTTGA
Il23A	51561	CTCAGGGACAACAGTCAGTTC	ACAGGGCTATCAGGGAGCA
CD80	941	AAACTCGCATCTACTGGCAAA	GGTTCTTGTACTCGGGCCATA
CD86	942	CTGCTCATCTATACACGGTTACC	GGAAACGTCGTACAGTTCTGTG
MMP9	4318	AGACCTGGGCAGATTCCAAAC	CGGCAAGTCTTCCGAGTAGT
IL10	3586	GACTTTAAGGGTTACCTGGGTTG	TCACATGCGCCTTGATGTCTG
TGFB1	7040	CAATTCCTGGCGATACCTCAG	GCACAACTCCGGTGACATCAA
CD163	9332	GCGGGAGAGTGGAAGTGAAAG	GTTACAAATCACAGAGACCGCT
NOS2	4843	AGGGACAAGCCTACCCCTC	CTCATCTCCCGTCAGTTGGT
CCL2	6347	CAGCCAGATGCAATCAATGCC	TGGAATCCTGAACCCACTTCT
MRC1	4360	CTACAAGGGATCGGGTTTATGGA	TTGGCATTGCCTAGTAGCGTA
TLR1	7096	TTCAAACGTGAAGCTACAGGG	CCGAACACATCGCTGACAACT
HLA-DRA	3122	TCTGGCGGCTTGAAGAATTTG	GGTGATCGGAGTATAGTTGGAGC
HLA-DRB	3123	CGGGGTTGGTGAGAGCTTC	AACCACCTGACTTCAATGCTG
CCL22	6367	ATTACGTCCGTTACCGTCTGC	TCCCTGAAGGTTAGCAACACC
CXCL10	3627	GAGCCTACAGCAGAGGAACC	GCTGATGCAGGTTACAGCGT
FN1	2335	GGTGGAATAGAGCTCCCAGG	GCAGCCTGCATCTGAGTACA
ACTB	60	AGAGCTACGAGCTGCCTGAC	AGCACTGTGTGTTGGCGTACAG

Supplementary Table 2. List of antibodies used in manuscript.

Target Protein/Antibody	Conjugation	Catalog Number	Source	Dilution	Clone Number	Lot Number
CLTC	-	ab21679	Abcam	1:1000	Polyclonal	64384377
TfR1	-	13113	Cell Signaling Technology	1:1000	D7G9X	2
CD81	-	56039	Cell Signaling Technology	1:1000	D3N2D	1
CD9	-	13174	Cell Signaling Technology	1:1000	D8O1	4
Flotillin-1	-	18634	Cell Signaling Technology	1:1000	D2V7J	1
Alix	-	92880	Cell Signaling Technology	1:1000	E6P9B	5
MSR1	-	17275	Cell Signaling Technology	1:1000	D8K4E	1
GM130	-	12480	Cell Signaling Technology	1:1000	D6B1	3
Annexin V	-	8555	Cell Signaling Technology	1:1000	Polyclonal	1
ICAM1	-	67836	Cell Signaling Technology	1:1000	E3Q9N	1
GAPDH	-	PA5-85074	Invitrogen	1:5000	Polyclonal	VB2950703B
Lamin B1	-	ab16048	Abcam	1:1000	Polyclonal	GR3188002-1
β-actin	-	66009-1	Proteintech	1:10,000	2D4H5	10021788
α-tubulin	-	3873	Cell Signaling Technology	1:1000	DM1A	12
Anti-rabbit IgG	HRP	7074	Cell Signaling Technology	1:10,000	Polyclonal	36
Anti-mouse IgG	HRP	7076	Cell Signaling Technology	1:10,000	Polyclonal	29
ICAM1	-	MA5407	Invitrogen	1:50	1A29	WD319948
Mouse IgG1 κ Isotype Control (P3.6.2.8.1)	-	16-4714-82	Invitrogen (eBioscience)	1:50	P3.6.2.8.1	Not available
CD204/MSR1	PE	371904	BioLegend	1:20	7C9C20	B336729
Mouse IgG2a, κ Isotype Control Antibody	PE	400214	BioLegend	1:20	MOPC-173	B342482
CD71/TfR1	APC	17-0719-41	Invitrogen (eBioscience)	1:20	OKT9	4331117
Mouse IgG1 κ Isotype Control Antibody (P3.6.2.8.1)	APC	17-4714-82	Invitrogen (eBioscience)	1:20	P3.6.2.8.1	2548810
CD115	Alexa Fluor 488	347312	BioLegend	1:20	9-4D2-1E4	B347763
CD86	PE	12-0862-82	Invitrogen (eBioscience)	1:100	GL1	Not available
HLA-DR	APC	17-9956-42	BioLegend	1:100	LN3	2350758
SIRPα (CD172a)	APC	372106	BioLegend	1:100	15-414	B341877
CD14	APC	325608	BioLegend	1:100	HCD14	B361508
CD11b	FITC	53-0112-82	Invitrogen (eBioscience)	1:100	M1/70	Not available
25F9	e660	50-0115-42	Invitrogen (eBioscience)	1:100	25F9	2446953
CD204	BV421	742438	Becton Dickinson	1:100	U23-56	2138404
CD280	PE	566817	Becton Dickinson	1:100	E1/183	2011749
EEA1	-	MA514794	Invitrogen	1:500	F.43.1	WD3260941
LAMP1	-	9091	Cell Signaling Technology	1:200	D2D11	7

Goat Anti-Rabbit IgG (H+L) Secondary Antibody	Alexa Fluor 647	A-21244	Invitrogen	1:500	Polyclonal	A21244
CD63	-	25682-1-AP	Proteintech	1:100	Polyclonal	Not available
Goat Anti-Rabbit IgG (H+L)	Alexa Fluor 568	A-11036	Invitrogen	1:500	Polyclonal	Not available
ICAM-1	APC	353112	BioLegend	1:100	HA58	B391968
CD11b	FITC	ab269333	Abcam	1:100	ICRF44	Not available
