# Supplementary material for: Harnessing macrophage-drug conjugates for allogeneic cell-based therapy of solid tumors via the TRAIN mechanism
Source: Nat Commun. 2025 Feb 4;16:1327. doi: 10.1038/s41467-025-56637-9 (PMC11790938; doi:10.1038/s41467-025-56637-9)
Supplement: Supplementary file 2 — Description of Additional Supplementary Files [file 41467_2025_56637_MOESM2_ESM.pdf]

Description of additional supplementary file

Supplementary Video 1

Time-lapse confocal microscopy video demonstrating the transfer of HFtFITC (Green) from BMDMs to EMT6 cancer cells (Red). The footage distinctly captures the dynamic process of HFt-FITC transfer during direct cell-cell interactions. Presented with a time format of HH:MM:SS:fff.

Supplementary Video 2

Time-lapse confocal microscopy video demonstrating the transfer of HFtFITC (Green) from BMDMs to EMT6 cancer cells (Red). The footage distinctly captures the dynamic process of HFt-FITC transfer during direct cell-cell interactions. Presented with a time format of HH:MM:SS:fff

Supplementary Video 3

Time-lapse confocal microscopy video demonstrating the transfer of HFtFITC (Green) from BMDMs to EMT6 cancer cells (Red). The footage distinctly captures the dynamic process of HFt-FITC transfer during direct cell-cell interactions. Presented with a time format of HH:MM:SS:fff.
