# Supplementary material for: Harnessing macrophage-drug conjugates for allogeneic cell-based therapy of solid tumors via the TRAIN mechanism
Source: Nat Commun. 2025 Feb 4;16:1327. doi: 10.1038/s41467-025-56637-9 (PMC11790938; doi:10.1038/s41467-025-56637-9)
Supplement: Supplementary file 6 — Reporting Summary [file 41467_2025_56637_MOESM6_ESM.pdf]

nature portfolio

Corresponding author(s): Magdalena Krol

Last updated by author(s): Nov 21, 2024

Reporting Summary

Nature Portfolio wishes to improve the reproducibility of the work that we publish. This form provides structure for consistency and transparency in reporting. For further information on Nature Portfolio policies, see our Editorial Policies and the Editorial Policy Checklist.

Statistics

For all statistical analyses, confirm that the following items are present in the figure legend, table legend, main text, or Methods section.

n/a	Confirmed	
☐	☒	The exact sample size (*n*) for each experimental group/condition, given as a discrete number and unit of measurement
☐	☒	A statement on whether measurements were taken from distinct samples or whether the same sample was measured repeatedly
☐	☒	The statistical test(s) used AND whether they are one- or two-sided<br>*Only common tests should be described solely by name; describe more complex techniques in the Methods section.*
☒	☐	A description of all covariates tested
☒	☐	A description of any assumptions or corrections, such as tests of normality and adjustment for multiple comparisons
☐	☒	A full description of the statistical parameters including central tendency (e.g. means) or other basic estimates (e.g. regression coefficient) AND variation (e.g. standard deviation) or associated estimates of uncertainty (e.g. confidence intervals)
☐	☒	For null hypothesis testing, the test statistic (e.g. *F*, *t*, *r*) with confidence intervals, effect sizes, degrees of freedom and *P* value noted<br>*Give P values as exact values whenever suitable.*
☒	☐	For Bayesian analysis, information on the choice of priors and Markov chain Monte Carlo settings
☒	☐	For hierarchical and complex designs, identification of the appropriate level for tests and full reporting of outcomes
☒	☐	Estimates of effect sizes (e.g. Cohen's *d*, Pearson's *r*), indicating how they were calculated

Our web collection on statistics for biologists contains articles on many of the points above.

Software and code

Policy information about availability of computer code

Data collection	In flow cytometry experiments, data was collected with BD FACSDiva™ Software (BD). Imaging flow cytometry data was collected with IDEAS 6.2 software (Amnis). Confocal microscopy images were acquired using LAS X (Leica) and FV10-ASW 3.0 (Olympus) software. Images in histology analysis were acquired using NIS-Elements Br-2 as software v 5.10 (Nikon). Luminesce images were captured with LivingImage software v. 4.3.1 (Caliper Life Sciences). Western blot images were quantified by using iBright Analysis software (Thermo Fisher Scientific). Absorbance in MTT tests was acquired with i-control software (Tecan). Real-Time PCR data was acquired with MxPro qPCR Software (Agilent). Absorbance of the ferritin complexes for protein concentration measurement was acquired with DS-11 Series software (DeNovix). UV-vis data was recorded with Nanodrop 2000 software v.1.4 (Thermo Fisher Scientific). Alphascreen signal was measured using SparkControl software (Tecan). Isothermal spectral shift measurements were carried out using MO.Control 2 software (NanoTemper).
Data analysis	GraphPad Prism 9.5.1 (GraphPad Software, Inc.), FlowJo v.10 (FlowJo LLC), FIJI software (ImageJ) and R v.4.2 (r-project.org) with packages 'survival', 'survminer' and 'ggplot2' used for data analysis

For manuscripts utilizing custom algorithms or software that are central to the research but not yet described in published literature, software must be made available to editors and reviewers. We strongly encourage code deposition in a community repository (e.g. GitHub). See the Nature Portfolio guidelines for submitting code & software for further information.

Data

Policy information about availability of data

All manuscripts must include a data availability statement. This statement should provide the following information, where applicable:

- Accession codes, unique identifiers, or web links for publicly available datasets
- A description of any restrictions on data availability
- For clinical datasets or third party data, please ensure that the statement adheres to our policy

Data for all main and supplementary figures can be obtained from the corresponding authors if requested.

Human research participants

Policy information about studies involving human research participants and Sex and Gender in Research.

Reporting on sex and gender	We collected PBMCs via leukapheresis from healthy volunteers of both sexes at the Institute of Hematology and Transfusion Medicine, Warsaw. Sex and gender data were not specifically analyzed or reported, as the focus was on isolating monocytes for downstream applications. As this information was not deemed relevant for the study design or the resulting data, no sex- or gender-based analysis was performed.
Population characteristics	PBMCs were obtained from healthy volunteers through leukapheresis. No data on age, sex, or other demographic factors were included in the study as the primary focus was the isolation of monocytes for large-scale production of hMDM used in in vivo studies.
Recruitment	Participants were recruited through the Institute of Hematology and Transfusion Medicine, Warsaw, following ethical approval from the Bioethics Committee of the Regional Medical Chamber in Warsaw. The leukapheresis procedure was carried out using the Spectra Optia system by MTZ Clinical Research Sp. z o.o. No specific inclusion or exclusion criteria related to sex or age were applied, as these were not anticipated to impact the results.
Ethics oversight	Bioethics Committee of the Regional Medical Chamber in Warsaw (study no. KB/1359/21)

Note that full information on the approval of the study protocol must also be provided in the manuscript.

Field-specific reporting

Please select the one below that is the best fit for your research. If you are not sure, read the appropriate sections before making your selection.

☒ Life sciences ☐ Behavioural & social sciences ☐ Ecological, evolutionary & environmental sciences

For a reference copy of the document with all sections, see nature.com/documents/nr-reporting-summary-flat.pdf

Life sciences study design

All studies must disclose on these points even when the disclosure is negative.

Sample size	Sample sizes were based on those reported in existing publications related to similar types of in vitro assays and in vivo therapeutic studies. Experiments were conducted in at least duplicate or triplicate. The chosen sample sizes were confirmed to be adequate through the significant and reproducible differences we consistently observed across groups.
Data exclusions	No relevant data were excluded.
Replication	All experiments were performed using at least two biological replicates, unless stated otherwise in the figure legends. Primary cells used in experiments were obtained from multiple donors. All attempts at replication were successful.
Randomization	Following the implantation of tumors, animals were randomized into different experimental groups.
Blinding	Blinding was implemented only in analysis of immunohistochemistry sections. All the other experiments were performed without blinding. This approach was partly due to practical considerations, as most experiments were carried out by a single investigator who was aware of experimental groups, making blinding unviable. Whenever possible, internal controls were implemented to facilitate quantification and analysis of data.

Reporting for specific materials, systems and methods

We require information from authors about some types of materials, experimental systems and methods used in many studies. Here, indicate whether each material, system or method listed is relevant to your study. If you are not sure if a list item applies to your research, read the appropriate section before selecting a response.

Materials & experimental systems

n/a	Involved in the study
☐	☒ Antibodies
☐	☒ Eukaryotic cell lines
☒	☐ Palaeontology and archaeology
☐	☒ Animals and other organisms
☒	☐ Clinical data
☒	☐ Dual use research of concern

Methods

n/a	Involved in the study
☒	☐ ChIP-seq
☐	☒ Flow cytometry
☒	☐ MRI-based neuroimaging

Antibodies

Antibodies used

Antibody, supplier name, catalog number, clone number, lot number:
1. anti-clathrin heavy chain, Abcam, ab21679, Polyclonal, 64384377
2. anti-TfR1, Cell Signaling Technology, 13113, D7G9X, 2
3. anti-CD81, Cell Signaling Technology, 56039, D3N2D, 1
4. anti-CD9, Cell Signaling Technology, 13174, D8O1, 4
5. anti-flotillin-1, Cell Signaling Technology, 18634, D2V7J, 1
6. anti-Alix, Cell Signaling Technology, 92880, E6P9B, 5
7. anti-GM130, Cell Singling Technology, 12480, D6B1, 3
8. anti-Annexin V, Cell Signaling Technology, 8555, Polyclonal, 1
9. anti-ICAM1, Cell Signaling Technology, 67836, E3Q9N, 1
10. anti-β-actin, Proteintech, 66009-1, 2D4H5, 10021788
11. anti-α-tubulin, Cell Signaling Technology, 3873T, DM1A, 12
12. anti-rabbit IgG, HRP-linked antibody, Cell Signaling Technology, 7074, Polyclonal, 36
13. anti-mouse IgG, HRP-linked antibody, Cell Signaling Technology, 7076, Polyclonal, 29
14. anti-CD206, Biolegend, 321104, 15.2, B343125
15. anti-CD86, BD Biosciences, 555658, 2331, 1187798
16. anti-CD163, BD Biosciences, 567881, MAC2-158, 1238541
17. anti-HLA-DR, Thermofisher Scientific, 17-9956-42, LN3, 2350758
18. anti-CD115, Biolegend, 347312, 9-4D2-1E4, B347763
19. anti-CD63, Proteintech, 25682-1-AP, Polyclonal, NA
20. anti-Rabbit IgG (H+L), Thermo Fisher, A-11036, Polyclonal, NA
21. anti-ICAM-1, BioLegend, 353113, HA58, B301338
22. anti-CD11b, Abcam, ab269333, ICRF44, NA
23. anti-MSR1, Cell Signaling Technology, 17275S, D8K4E, 1
24. anti-GAPDH, Invitrogen, PA5-85074, Polyclonal, VB2950703B
25. anti-lamin B1, Abcam, ab16048, Polyclonal, GR3188002-1
26. anti-ICAM-1-APC, BioLegend, 353112, HA58, B391968
27. anti-CD11b FITC, Invitrogen, 53-0112-82, M1/70,
28. anti-CD68, Cell Signaling Technology, 26042, D4B9C, NA
29. anti-Ki67, Cell Signaling Technology, 34330, Ki-67, NA
30. anti-EEA1, Invitrogen, MA514794, F.43.1, WD3260941
31. anti-LAMP1, Cell Signaling Technology, 9091S, D2D11, 7
32. anti-ICAM-1, Invitrogen, MA5407, 1A29, WD319948
33. Mouse IgG1 isotype control, Invitrogen, 14-4714-85 , P3.6.2.8.1, 2504991
34. PE mouse IgG2a, κ isotype control antibody, BioLegend, #400214, MOPC-173, B342482
35. APC mouse IgG1 κ isotype control antibody, eBioscience, #17-4714-82, P3.6.2.8.1, 2548810
36. anti-CAV1, Cell Signaling Technology, 3267S, 9
37. anti-CD204/MSR1, BioLegend, 371904, 7C9C20, B336729
38. anti-CD86, Invitrogen (eBioscience), 12-0862-82, GL1, NA
39. anti-SIRPα (CD172a), BioLegend, 372106, 15-414, B341877
40. anti-CD14, BioLegend, 325608, HCD14, B361508
41. anti-25F9, Invitrogen (eBioscience), 50-0115-42, 25F9, 2446953
42. anti-CD204, Becton Dickinson, 742438, U23-56, 2138404
43. anti-CD280, Becton Dickinson, 566817, E1/183, 2011749
44. goat anti-Rabbit IgG (H+L) Secondary Antibody, Invitrogen, A-21244, Polyclonal, A21244
45. anti-CD71/TfR1, Invitrogen (eBioscience), 17-0719-41, OKT9, 4331117

Validation

Validation statements for all antibodies listed above can be found through the following links to the manufacturer's website.
1. https://www.abcam.com/en-pl/products/primary-antibodies/clathrin-heavy-chain-antibody-ab21679
2. https://www.cellsignal.com/products/primary-antibodies/cd71-d7g9x-xp-rabbit-mab/13113
3. https://www.cellsignal.com/products/primary-antibodies/cd81-d3n2d-rabbit-mab/56039
4. https://www.cellsignal.com/products/primary-antibodies/cd9-d8o1a-rabbit-mab/13174
5. https://www.cellsignal.com/products/primary-antibodies/flotillin-1-d2v7j-xp-rabbit-mab/18634
6. https://www.cellsignal.com/products/primary-antibodies/alix-e6p9b-rabbit-mab/92880
7. https://www.cellsignal.com/products/primary-antibodies/gm130-d6b1-xp-rabbit-mab/12480
8. https://www.cellsignal.com/products/primary-antibodies/annexin-v-antibody/8555

9. https://www.cellsignal.com/products/primary-antibodies/cd54-icam-1-e3q9n-xp-rabbit-mab/67836
10. https://www.ptglab.com/products/Pan-Actin-Antibody-66009-1-Ig.htm
11. https://www.cellsignal.com/products/primary-antibodies/a-tubulin-dm1a-mouse-mab/3873
12. https://www.cellsignal.com/products/secondary-antibodies/anti-rabbit-igg-hrp-linked-antibody/7074
13. https://www.cellsignal.com/products/secondary-antibodies/anti-mouse-igg-hrp-linked-antibody/7076
14. https://www.biolegend.com/en-gb/explore-new-products/fitc-anti-human-cd206-mmr-antibody-2993?GroupID=BLG8520
15. https://www.bdbiosciences.com/en-au/products/reagents/flow-cytometry-reagents/research-reagents/single-color-antibodies-ruo/pe-mouse-anti-human-cd86.555658
16. https://www.bdbiosciences.com/en-es/products/reagents/flow-cytometry-reagents/research-reagents/single-color-antibodies-ruo/pe-mouse-anti-human-cd163.567881
17. https://www.thermofisher.com/antibody/product/HLA-DR-Antibody-clone-LN3-Monoclonal/17-9956-42
18. https://www.biolegend.com/en-gb/products/alexa-fluor-488-anti-human-cd115-csf-1r-antibody-12825
19. https://www.ptglab.com/products/CD63-Antibody-25682-1-AP.htm
20. https://www.thermofisher.com/antibody/product/Goat-anti-Rabbit-IgG-H-L-Highly-Cross-Adsorbed-Secondary-Antibody-Polyclonal/A-11036
21. https://www.biolegend.com/en-us/products/alexa-fluor-647-anti-human-cd54-antibody-14743
22. https://www.abcam.com/en-pl/products/primary-antibodies/fitc-cd11b-antibody-icrf44-ab269333#support
23. https://www.cellsignal.com/products/primary-antibodies/msr1-d8k4e-rabbit-mab/17275
24. https://www.thermofisher.com/antibody/product/GAPDH-Antibody-Polyclonal/PA5-85074
25. https://www.abcam.com/en-us/products/primary-antibodies/lamin-b1-antibody-nuclear-envelope-marker-ab16048
26. https://www.biolegend.com/fr-lu/products/apc-anti-human-cd54-antibody-7484
27. https://www.thermofisher.com/antibody/product/CD11b-Antibody-clone-M1-70-Monoclonal/53-0112-82
28. https://www.cellsignal.com/products/primary-antibodies/cd68-d4b9c-xp-rabbit-mab-bsa-and-azide-free/26042
29. https://www.cellsignal.com/products/primary-antibodies/ki-67-d3b5-rabbit-mab-bsa-and-azide-free/34330
30. https://www.thermofisher.com/antibody/product/EEA1-Antibody-clone-F-43-1-Monoclonal/MA5-14794
31. https://www.cellsignal.com/products/primary-antibodies/lamp1-d2d11-xp-rabbit-mab/9091
32. https://www.thermofisher.com/antibody/product/ICAM-1-Antibody-clone-1A29-Monoclonal/MA5407
33. https://www.thermofisher.com/order/genome-database/generatePdf?productName=Mouse%20IgG1%20kappa&assayType=PRANT&detailed=true&productId=14-4714-85
34. https://www.biolegend.com/ja-jp/products/pe-mouse-igg2a-kappa-isotype-ctrl-fc-3043
35. https://www.thermofisher.com/antibody/product/Mouse-IgG1-kappa-clone-P3-6-2-8-1-Monoclonal/17-4714-82
36. https://www.cellsignal.com/products/primary-antibodies/caveolin-1-d46g3-xp-rabbit-mab/3267?srsltid=AfmBOorfX17vAdOJoZIIDk9fp36On0g3ERHHBGOSZBomvwBAyuGCkIbv
37. https://www.biolegend.com/en-gb/products/pe-anti-human-cd204-antibody-14131
38. https://www.thermofisher.com/antibody/product/CD86-B7-2-Antibody-clone-GL1-Monoclonal/12-0862-82
39. https://www.biolegend.com/de-de/products/apc-anti-human-cd172a-sirpalpha-antibody-14165?GroupID=BLG15632
40. https://www.biolegend.com/de-de/products/apc-anti-human-cd14-antibody-3953
41. https://www.thermofisher.com/antibody/product/Mature-Macrophage-Marker-Antibody-clone-eBio25F9-25F9-Monoclonal/50-0115-42
42. https://www.bdbiosciences.com/en-pl/products/reagents/flow-cytometry-reagents/research-reagents/single-color-antibodies-ruo/bv421-mouse-anti-human-msr1-cd204.742438
43. https://www.bdbiosciences.com/en-pl/products/reagents/flow-cytometry-reagents/research-reagents/single-color-antibodies-ruo/pe-mouse-anti-human-cd280-mrc2.566817
44. https://www.thermofisher.com/antibody/product/Goat-anti-Rabbit-IgG-H-L-Cross-Adsorbed-Secondary-Antibody-Polyclonal/A-21244
45. https://www.thermofisher.com/antibody/product/CD71-Transferrin-Receptor-Antibody-clone-OKT9-OKT-9-Monoclonal/17-0719-41

Eukaryotic cell lines

Policy information about cell lines and Sex and Gender in Research

Cell line source(s)

Cell lines obtained from ATCC:
human leukemia monocytic cell line THP-1 (ATCC® TIB-202™)
human breast cancer cell line MDAMB231 (ATCC® HTB-26™)
human colon cancer cell line LoVo (ATCC® CCL-229™)
human embryonic kidney cell line HEK-293 (ATCC® CRL-1573™)
Chinese hamster ovary cell line CHO-K1 (ATCC® CCL-61™)
human glioblastoma cell line U-87 MG (ATCC® HTB-14)
murine macrophage cell line RAW 264.7 (ATCC® TIB-71™)
murine mammary cancer cell line EMT6 (ATTC® CRL-2755™)
murine mammary cancer cell line 4T1 (ATTC® CRL 2539™)
human colon cancer cell line Caco2 (ATCC® HTB-37™)
human colon cancer cell line Colo205 (ATCC® CCL-222™)
human colon cancer cell line DLD1 (ATCC® CCL-221™)
human colon cancer cell line HT-29 (ATCC® HTB-38™)
human colon cancer cell line SW480 (ATCC® CCL-228™)
human prostate cancer cell line LNCaP (ATCC® CRL-1740™)
human lung cancer cell line A549 (ATCC® CCL-185™)
human chondrosarcoma cell line SW 1353 (ATCC® HTB-94™)
human pancreas cancer cell line BxPC3 (ATCC® CRL-1687™)
primary human bladder fibroblast cells (ATCC® PCS-420-013)
primary human renal proximal tubule epithelial cells (ATCC® PCS-400-010)
human lung fibroblast MRC-5 cells (ATCC® CCL-171)

Cell lines kindly provided by the Medical University of Warsaw:
murine colon cancer cell line CT26.WT (ATCC® CRL-2638™)
human cervical cancer cell line HeLa (ATCC® CCL-2™)
human ovarian cancer cell line SKOV3 (ATCC® HTB-77™)
human breast cancer cell line MCF7 (ATCC® HTB-22™)
human pancreatic cancer cell line HPAC (ATCC® CRL-2119™)
human colon cancer cell line HCT 116 (ATCC® CCL-247™)
human prostate cancer cell line DU 145 (ATCC® HTB 81™)
murine connective mouse tissue L929 (ATCC® CCL-1™)

Cell lines provided by Axis Bio Services Ltd.:
murine bladder carcinoma MB49
murine head and neck carcinoma SCC7

Cell lines kindly provided by the Maria Skłodowska Curie Memorial Cancer Centre and Institute of Oncology (MCMCC) in Warsaw:
human colon cancer cell line CL-11 (DSMZ® ACC 467)
human colon cancer cell line CL-40 (DSMZ® ACC 535)
Other sources:
mouse glioma cell line GL261 (Division of Cancer Treatment and Diagnosis (DCTD) Tumor Repository, National Cancer Institute)
murine mammary cancer cell line EMT6_Fluc-Puro (Imanis Life Sciences, CL154)

PBMCs were isolated from buffy coats obtained from anonymous healthy donors of both sexes purchased from the Regional Blood Donation and Blood Treatment Center in Warsaw, Poland.
Murine bone marrow cells for bone marrow-derived macrophages were obtained from female BALB/c mice.
Human induced pluripotent stem-cell (iPSC) line SFCi55 (CVCL_UL47) was generated from fibroblasts from blood group O Rhesus negative female donor.

Authentication	The cell lines used were not authenticated beyond the identity information provided by ATCC.
Mycoplasma contamination	All cell lines tested negative for mycoplasma contamination.
Commonly misidentified lines (See ICLAC register)	No commonly misidentified cell lines were used.

Animals and other research organisms

Policy information about studies involving animals; ARRIVE guidelines recommended for reporting animal research, and Sex and Gender in Research

Laboratory animals

Female athymic nude mice aged 6-8 weeks were used for the study with MDC-250. These were bred in-house by Axis Bio (Ireland) therefore they required no acclimatization period. Animals were housed in IVC cages (up to 5 per cage) with individual mice identified by tail mark. All animals were allowed free access to a standard certified commercial diet and sanitized water during the study. The holding room was maintained under standard conditions: 20-24 °C, 40-70% humidity, and a 12h light/dark cycle. Each cage was subjected to 51-54 air changes per hour and the holding room had 14 air changes per hour.

BALB/C mice used as bone marrow donors and for MDC-Dox therapeutic experiments were obtained from Animalab (Poznań, Poland), which supplies mice bred by Charles River Laboratories. Female BALB/C mice aged 6-8 weeks were housed in IVC cages (up to 5 per cage). All animals were allowed free access to a standard certified commercial diet and sanitized water during the study. The holding room was maintained under standard conditions: 20-24 °C, 40-70% humidity, and a 12h light/dark cycle. Each cage was subjected to 51-54 air changes per hour and the holding room had 14 air changes per hour.

A total of 52 female athymic nude mice aged 6-8 weeks were used for the study with MDC-250 in the BxPC-3_luc model. These were bred in-house by Axis Bio Services Ltd., requiring no acclimatization period. The animals were housed in IVC cages (up to 5 per cage), and each mouse was identified by tail mark. They had free access to a standard certified commercial diet and sanitized water throughout the study. The holding room was maintained at 20-24°C with 40-70% humidity and a 12-hour light/dark cycle, with 51-54 air exchanges per hour per cage and 14 air changes per hour in the room.

A total of 32 female athymic nude mice (aged 6-8 weeks) were used for the MDC-735 study in the BxPC-3_luc model. These were bred in-house by Axis Bio Services Ltd. with no acclimatization period required. The animals were housed in IVC cages (up to 5 per cage) and identified by tail marks. Standard environmental conditions were maintained, with the room at 18-24°C, humidity between 55-70%, and a 12-hour light/dark cycle. Mice were provided free access to a certified diet and sanitized water.

A total of 24 female BALB/C mice aged 6-8 weeks purchased from AnimalLab were used for the study with MDC-Dox in the EMT6_luc model. The animals were housed in IVC cages (up to 5 per cage) under standard conditions of 20-24°C, 40-70% humidity, and a 12-hour light/dark cycle. Free access to a standard certified commercial diet and sanitized water was provided.

A total of 40 female C3H/HeN mice (aged 5-8 weeks, 22-30 g) were purchased from Charles River and acclimatized for 7 days prior to the study. The animals were housed in IVC cages (up to 5 per cage) under standard conditions of 20-24°C, 30-70% humidity, and a 12-hour light/dark cycle.

A total of 40 male C57BL/6 mice (aged 6-8 weeks) were purchased from Janvier Laboratories for use in the MB49 tumor model. The

	animals were housed under standard conditions (20-24°C, 30-70% humidity, and a 12-hour light/dark cycle) with free access to a certified commercial diet and sanitized water.<br><br>A total of 28 female athymic nude mice (aged 5-7 weeks) were purchased from Charles River, UK, and acclimatized for 7 days before the study with MDC-735 in the SK-OV-3 model. Mice were housed in IVC cages (up to 5 per cage) under standard conditions of 20-24°C, 40-70% humidity, and a 12-hour light/dark cycle.<br><br>A total of 50 female Balb/c mice (aged 6-8 weeks) were purchased from AnimalLab and acclimatized for 7 days before the start of the MDC-735 study in the EMT6_Fluc lung colonization model. Mice were housed under standard conditions (20-24°C, 40-70% humidity, and a 12-hour light/dark cycle) with 51-54 air changes per hour per cage.
Wild animals	The study did not involve wild animals.
Reporting on sex	Only female mice were used in the experimental design.
Field-collected samples	The study did not involve samples collected from the field.
Ethics oversight	Protocols for animal experiment with athymic nude mice have been approved by the Axis Bioservices Animal Welfare and Ethical Review Committee, and all procedures were carried out under the guidelines of the Animal (Scientific Procedures) Act 1986. Protocols for animal experiment with BALB/C mice have been approved by the 1st Local Ethical Committee for Experiments on Animals, Warsaw, Poland.

Note that full information on the approval of the study protocol must also be provided in the manuscript.

Flow Cytometry

Plots

Confirm that:

☒ The axis labels state the marker and fluorochrome used (e.g. CD4-FITC).

☒ The axis scales are clearly visible. Include numbers along axes only for bottom left plot of group (a 'group' is an analysis of identical markers).

☒ All plots are contour plots with outliers or pseudocolor plots.

☒ A numerical value for number of cells or percentage (with statistics) is provided.

Methodology

Sample preparation	Detailed sample preparation is described in the Methods section. Adherent cells were detached using Accutase (Sigma Aldrich) or TrypLE Express (Gibco) and transferred to 5 ml round-bottom polystyrene tubes for further processing. Cells were washed with PBS before and after each staining protocol. In experiments with cells expressing Fc receptor, Fc block (Miltenyi Biotech) was used according to the manufacturers protocol. Staining with antibodies was performed by incubation at predetermined optimum concentrations at 4 °C for 20min. Prior to analysis, cells were resuspended in flow cytometry buffer in the presence of DRAQ7 (Invitrogen) or Zombie (Biolegend) Dead Cell Stain then samples were acquired using BDAria II or BD Canto II flow cytometer.<br>In co-culture experiments, CellTrace cytoplasmic dyes (Thermo Fisher Scientific) were used to distinguish cell populations. In in vitro killing assays in co-culture CountBright™ Absolute Counting Beads (Invitrogen) were added prior to cell acquisition to determine the viability of cancer cells as a number of live cells relative to a control condition.<br>In imaging flow cytometry experiments, single-color controls were prepared to create compensation matrix according to the IDEAS recommended sample analysis protocol.<br>In phenotyping flow cytometry experiments, single-color controls were prepared to create compensation and FMO controls for analysis.
Instrument	Flow cytometry acquisition was performed using BDAria II or BD Canto II flow cytometer. Imaging flow cytometry was performed using Amnis ImageStream X.
Software	FACS Diva was used for flow cytometry data collection, IDEAS software was used for imaging flow cytometry data collection. FlowJo and IDEAS software were used for data analysis, respectively.
Cell population abundance	RAW264.7 transfected with GFP-C1-PLCdelta-PH plasmid, were sorted based on GFP fluorescence intensity using a FACS Aria II sorter. To verify the sorting process, the purity of the sorted populations was assessed by conducting a post-sort purity check.

Gating strategy

For all samples, the gating strategy began with the use of forward scatter (FSC) and side scatter (SSC) to identify cell events. This was followed by forward scatter height versus area gating to select single cells. Live cells were then identified based on their lack of staining with a viability dye. Appropriate negative controls were used at each stage to accurately define the gates of interest.
In co-culture experiments, CellTrace dyes were used to distinguish cell populations, prior to single cell gating. CountBright™ Absolute Counting Beads (Invitrogen) were gated according to manufacturers manual.

☒ Tick this box to confirm that a figure exemplifying the gating strategy is provided in the Supplementary Information.
